# Identification of 17 HrpX-Regulated Proteins Including Two Novel Type III Effectors, XOC_3956 and XOC_1550, in *Xanthomonas oryzae* pv. *oryzicola*

**DOI:** 10.1371/journal.pone.0093205

**Published:** 2014-03-27

**Authors:** Xiao-bo Xue, Li-fang Zou, Wen-xiu Ma, Zhi-yang Liu, Gong-you Chen

**Affiliations:** 1 School of Agriculture and Biology, Shanghai Jiao Tong University/Key Laboratory of Urban (South) by Ministry of Agriculture, Shanghai, China; 2 State Key Laboratory of Microbial Metabolism, School of Life Science and Biotechnology, Shanghai Jiao Tong University, Shanghai, China; Universidad Nacional de La Plata., Argentina

## Abstract

The function of some hypothetical proteins, possibly regulated by key *hrp* regulators, in the pathogenicity of phytopathogenic bacteria remains largely unknown. In the present study, *in silicon* microarray data demonstrated that the expression of 17 HrpX-regulated protein (Xrp) genes of *X. oryzae* pv. *oryzicola* (*Xoc*), which causes bacterial leaf streak in rice, were either positively or negatively regulated by HrpX or/and HrpG. Bioinformatics analysis demonstrated that five Xrps possess a putative type III secretion (T3S) signal in the first 50 N-terminal amino acids, six *xrp* genes contain a PIP-box-like sequence (TTCGB-N_X_-TTCGB, 9≤X≤25) in the promoter regions, and two Xrps have both motifs. Twelve Xrps are widely conserved in *Xanthomonas* spp., whereas four are specific for *X. oryzae* (Xrp6) or *Xoc* (Xrp8, Xrp14 and Xrp17). In addition to the regulation by HrpG/HrpX, some of the 17 genes were also modulated by another *hrp* regulator HrpD6. Mutagenesis of these 17 genes indicated that five Xrps (Xrp1, Xrp2, Xrp5, Xrp8 and Xrp14) were required for full virulence and bacterial growth *in planta*. Immunoblotting assays and fusion with N-terminally truncated AvrXa10 indicated that Xrp3 and Xrp5 were secreted and translocated into rice cells through the type-III secretion system (T3S), suggesting they are novel T3S effectors. Our results suggest that *Xoc* exploits an orchestra of proteins that are regulated by HrpG, HrpX and HrpD6, and these proteins facilitate both infection and metabolism.

## Introduction

Bacterial leaf streak (BLS) of rice, which is caused by *Xanthomonas oryzae* pv. *oryzicola* (*Xoc*), is a destructive plant disease in Asia. The pathogen infects rice through leaf stomata or wounds and colonizes intercellular spaces in the mesophyll, resulting in water-soaked interveinal lesions that develop into translucent streaks [1]. The infection routes and the symptoms caused by *Xoc* differ from those incited by the closely-related pathogen, *X. oryzae* pv. *oryzae* (*Xoo*). *Xoo* enters rice leaves through hydathodes or wounds, propagates in the intercellular spaces of the underlying epidermis, and then spreads throughout the plant in the xylem, where it presumably interacts with xylem parenchyma cells [2], [3], [4]. The *Xoc*-rice pathosystem is an important working model to elucidate how pathogens evade the plant host immune system [2], [5]. The complete genome sequence and comparative and functional genomic studies of three *Xoo* strains like KACC10331 [6], PXO99^A^ [7], MAFF311018 [8] and *Xoc* strain BLS256 [9] have furthered our understanding of *Xanthomonas*-rice interactions. However, it remains unclear whether the numerous hypothetical proteins, maybe possibly regulated by HrpX (Xrps) annotated in *X. oryzae*, are involved in virulence.

The type III secretion system (T3S) is a pathogenicity determinant machine for Gram-negative pathogenic bacteria in host plants [10], [11]. The Xanthomonad T3S is encoded by the *hrp-hrc-hpa* genes [5], [12] and secretes a repertoire of effector proteins (T3SEs) into plant cells to trigger plant disease development [13]–[16]. These T3SEs may function to overcome PAMP- (pathogen-associated molecular pattern) triggered immunity (PTI) and Effector-triggered immunity (ETI), or promote effector-triggered susceptibility (ETS) [17]–[19]. In *X. oryzae*, T3SEs are classified into two types: transcriptional activator-like effectors (TALEs) [6], [20], [21] and NTALEs (non-TAL effectors); the latter group is also known as the Xop (*Xanthomonas* out protein) effectors [16], [22]. Some of the NTALEs are Xrps originally annotated in the genomes of *Xanthomonas* spp. [16], implying that some Xrps may be uncharacterized T3SEs.

The expression of genes coding for the T3S and effectors is generally plant-inducible and regulated by a key *hrp* regulatory factor, HrpX [12], [14], [23], [24]. HrpX is an AraC-type transcriptional regulator that controls the expression of genes in the HrpX regulon by binding the PIP (plant-inducible promoter)-box; this is a conserved *cis*-element with the consensus TTCGB-N_15_-TTCGB (‘B’ refers to any base except adenine) [25]–[28]. The PIP-box is normally followed by a −10 box that is located at 30–32 bp further downstream [29]. T3SEs also contain secretion signals in the first 50 N-terminal amino acids, which are characterized by one or more of the following: ≥20% Ser and Pro [22], [26], [30]; more than five Ser residues [13], [31], [32]; an aliphatic amino acid (Ile, Leu, or Val) or Pro at the third or fourth position; and a lack of negatively charged amino acids within the first 12 residues [33]. However, it is important to note that genes in the HrpX regulon may not be T3SEs; for example, HrpX-regulated proteins from *Xoo* recently identified by 2D-difference gel electrophoresis (2-DIGE) did not function as T3SEs [34]. The transcription and translocation of HrpX regulon candidates have been examined using several reporter systems, such as calmodulin-dependent adenylate cyclase (Cya) of *Bordetella pertussis* [22], [30], [31], [35], *gusA* [36]–[40] and avirulence proteins (e.g., AvrBs1 and AvrXa10) lacking the T3S signal sequence [24], [38], [41].

The expression of HrpX is regulated by HrpG, which belongs to the OmpR family of two-component signal transduction response regulators [42], [43]. HrpG regulates the expression of *hrpX* and *hrpA* operon and also controls the expression of several proteins that function as cell wall degrading enzymes (CWDEs), which are secreted by the type II secretion system (T2SS) [14], [44], [45]. Recently, a novel *hrp* regulator, HrpD6, was identified and shown to be regulated by HrpG and HrpX; HrpD6 regulates the expression of *hpa2*, *hpa1*, *hpaB*, *hrcC*, and *hrcT* [12]. However, it remains unclear whether Xrps in *Xanthomonas* are regulated by HrpG or HrpD6.

In this study, bioinformatic and genetic approaches were used to characterize 17 Xrp-coding genes from *in silicon* data. Different transcriptional profiles of these genes in the wild-type strain *Xoc* RS105, *hrpG* (RΔ*hrpG*), and *hrpX* (RΔ*hrpX*) mutants [12] were compared. Two Xrp proteins, XOC_3956 and XOC_1550, were identified as new T3SEs in *Xoc*.

## Materials and Methods

### Bacterial strains, plasmids and growth conditions

The bacterial strains and plasmids used in this study are listed in Table S1 in File S1. *Escherichia coli* was grown at 37°C in Luria-Bertani medium [46]. *Xanthomonas* strains and other derivatives were grown in NB, NA, NAN, NAS [47], XOM3 [48] or with rice suspension cells [48]. Antibiotics were added at the following concentrations (µg/ml) when required: kanamycin (Kan), 25; rifampicin (Rif), 50; ampicillin (Ap), 100; and spectinomycin (Sp), 50.

### Microarray design

An oligonucleotide microarray was designed at the Shanghai Biotechnology Corporation (Shanghai, China). Each slide contained six arrays, and each array contained approximately 15,000 spots (our probes were represented in triplicate). For *Xoc* BLS256, the genome sequence was also available from the NCBI database as accession AAQN01000001(GI:94721269). Up to five candidate probes per target (sense orientation) were designed with the Agilent eArray web tool, using temperature-matching methodology, a preferred probe melting temperature of 80°C, no 3′bias, and a target length of 60 bp. Shorter probes were extended to 60 bp using the Agilent linker.

### RNA isolation and microarray execution

*Xoc* strain RS105 and the *hrpG* and *hrpX* mutants (RΔ*hrpG* and RΔ*hrpX*, respectively) [12] were cultured overnight in NB broth at 28°C in a shaking incubator and collected the following day via centrifugation at 5,000 rpm. Each strain was washed once in rice suspension cells [49] before resuspension in rice cells at OD_600_ = 0.6. Strains were then incubated for 16 h at 60 rpm at 25°C. RNA was extracted from 1 ml of co-cultivated cells using TRIzol® Reagent (Invitrogen, Shanghai, China) as described by the manufacturer. All RNA was quantified using an Eppendorf BioSpectrometer kinetic (Eppendorf, Shanghai, China) and checked for quality using an RNA 6000 Nano Kit and a 2100 Bioanalyzer (Agilent Technologies). Fluorescent labeling of total RNA was performed as described previously [49] using the following array design on a single 6×15 k format slide: 1, WT rep 1 (Cy3) and RΔ*hrpG* rep 1 (Cy5); 2, RΔ*hrpG* rep 2 (Cy3) and WT rep 2 (Cy5); 3, WT rep 3 (Cy3) and RΔ*hrpG* rep 3 (Cy5); 4, WT rep 1 (Cy3) and RΔ*hrp*X rep 1 (Cy5); 5, RΔ*hrpX* rep 2 (Cy3) and WT rep 2 (Cy5); 6, WT rep 3 (Cy3) and RΔ*hrpX* rep 3 (Cy5). This design incorporated a dye-swap and balanced labeling of all samples. Levels and efficiencies of labeling were estimated using a spectrophotometer. Microarray hybridization, washing and scanning were performed in the JHI Sequencing and Microarray Facility as described previously [50]. Microarray images were imported into Agilent Feature Extraction (FE) (v.9.5.3) software and aligned with the appropriate array grid template file (021826_D_F_20081029). Intensity data and quality control (QC) metrics were extracted using the recommended FE protocol (GE2-v5_95_Feb07). Entire FE datasets for each array were loaded into GeneSpring (v.7.3) software for further analysis.

### Microarray analysis

Data were normalized using default settings for two-channel arrays and transformed to account for dye-swaps. Data from each array were normalized using the Lowess algorithm to minimize differences in dye incorporation efficiency. Unreliable data flagged as absent in all replicate samples by the FE software were discarded. Gene lists with significant change were generated from combined replicate datasets for each pares, RΔ*hrpG*/RS105 and RΔ*hrpX*/RS105, using volcano plot filtering if the ratio is lower than 0.55 or higher than 1.75 with the *P* value less than 0.05 (Student's *t* test).

### DNA manipulation and plasmid construction

DNA manipulation was performed following standard procedures [46]. Biparental conjugal transfer of plasmids from *E. coli* to *Xoc* was performed as described previously [51]. PCR amplification was performed with primers (Table S2 in File S1) and genomic DNA of *Xoc* RS105; the genome sequence of *Xoc* BLS256 was used as a reference (http://cmr.jcvi.org/cgi-bin/CMR/GenomePage.cgi?org=Xoc). All plasmids constructs were confirmed by restriction enzyme digestion and sequencing.

To construct transcriptional fusions of *xrp* genes with the promoterless *gusA* (β-glucuronidase) gene, a 500-bp region upstream of each *xrp* ORF was PCR-amplified with primer sets p*xrpX*-F/p*xrpX*-R (X refers to 17 *xrp* genes; 1 to 17) (Table S2 in File S1). The PCR products were fused in-frame with *gusA* in pUFR034GUS (Table S1 in File S1), resulting in p*xrpX*GUS (X refers to 17 *xrp* genes; 1 to 17). Recombinant plasmids were introduced into the wild-type strain RS105, and the *hrpG*, *hrpX* and *hrpD6* mutants (see reference 12 for RΔ*hrpG*, RΔ*hrpX*, and RΔ*hrpD6*) (Table S1 in File S1) using biparental conjugation as described above.

To generate transcriptional fusions of *xrp* genes with c-Myc tags, fragments containing native promoters and corresponding *xrp* ORFs were PCR-amplified with the *xrpX*-F/*xrpX*-R primer pairs (X refers to 17 *xrp* genes, Table S2 in File S1) using RS105 genomic DNA as the template. After sequencing for confirmation, PCR products were cloned in pUFR034Myc in-frame at suitable enzyme sites, generating pXrpXMyc (X refers 1 to 17 xrp genes) (Table S1 in File S1). The recombinant plasmids were introduced into wild-type RS105 and the *hrcV* mutant (RΔ*hrcV*) by the biparental conjugation method.

Chimeric fusions were also generated between a variant of AvrXa10 lacking 28 amino acids at the N-terminus and *xrp3* and *xrp5*. The truncated form of AvrXa10 was designated AvrXa10Δ28 and has been described previously [26]. The sequences obtained from *xrp3* and *xrp5* included approximately 500 bp upstream of the translational start site and the first 150 nucleotides. These regions of *xrp3 and xrp5* were obtained using primer pairs *xrp3*-N-F/*xrp3*-N-R and *xrp5*-N-F/*xrp5*-N-R, respectively (Table S2 in File S1). The PCR products were ligated into pBlueAvrXa10Δ28 at *Sac*Ι and *Pst*Ι sites. The constructs were then cloned into pHMI at the *Sac*Ι site, resulting in pXrp3AvrXa10Δ28 and pXrp5AvrXa10Δ28 (Table S1 in File S1). Recombinant plasmids were introduced into *Xoo* PXO99^A^ and PΔ*hrcU* by electroporation.

YFP was used as a reporter to investigate the subcellular localization of selected T3SEs. The complete ORFs encoding *xrp3* and *xrp5* without stop codons were PCR-amplified from genomic DNA of *Xoc* RS105 with *xrp*3-Y-F/*xrp*3-Y-R and *xrp5*-Y-F/*xrp5*-Y-R, respectively (Table S2 in File S1). The products were cloned in-frame with *yfp* in pA7-YFP [52], resulting in pXrp3YFP and pXrp5YFP (Table S1 in File S1), respectively. These two recombinants were then transferred into *Arabidopsis* (Ecotype *Col-0*) mesophyll protoplasts by PEG-calcium fusion as described previously [53].

### Quantitative real-time PCR (qRT-PCR)

The expression of selected *xrp* genes was assayed by qRT-PCR with corresponding primer pairs (Table S2 in File S1). All primers were designed with Beacon Designer 7 software. RNA was extracted from *Xanthomonas* strains as described previously [47] using TRIzol® Reagent (Takara, Dalian, China) and the manufacturer's recommendations. cDNA synthesis was conducted with AMV random primers purchased from Takara. Prior to synthesis of the first-strand cDNA, total RNAs were digested with RNase-free DNase I (TaKaRa) to remove potential traces of genomic DNAs. qRT-PCR was performed on the Applied Biosystems 7500 qRT-PCR System using SYBR Premix ExTaq™ (Takara). PCR conditions included the following parameters: denaturation at 95°C for 30 s; 40 cycles at 95°C, 5 s; and 60°C, 34 s. Experiments were performed at least three times in triplicate. Internal controls included *gyrB* (*XOC_0006*, DNA gyrase B subunit) and *rpoD* (*XOC_2329*, RNA polymerase sigma factor 70) [28], [54].

### β-glucuronidase (GUS) activity assays

For GUS activity assays, *Xoc* strains were preincubated in 5 ml NB broth at 28°C for 16–20 h until the OD_600_ = 0.6. An aliquot (100 µl) of this culture was transferred into 5 ml fresh NB broth. Bacterial cells were collected, washed twice, and resuspended in XOM3 to an OD_600_ = 2.0. After incubation at 28°C for 6 h, 1 ml of sonic buffer (40 mM Tris-HCl, pH 7.0, 20 mM β-mercaptoethanol, 10 mM EDTA, and 2% Triton X-100) was added into 1 ml of the bacterial culture. This mixture was frozen in liquid nitrogen and then thawed in at 37°C for 5 min. This procedure was repeated five times, and the mixture was centrifuged (12,000 rpm) at 4°C for 15 min. Then, 90 µl 4-methylumbelliferone-β-glucuronide (4-MUG) (Sigma, Shanghai, China) [55] was added into 10 µl supernatant and incubated at 37°C for 1 h. The reaction was terminated by adding 1 ml of 2 M Na_2_CO_3_. GUS activity was measured at 415 nm with the Modulus™ Single Tube Multifunction Tester (YuanPingHao, Beijing, China) [56]. One unit was defined as 1 nM of 4-methyl-umbelliferone (4-MU) produced per min per OD_600_ of bacterial cells as described [56]. Three independent experiments were performed, and similar results were obtained.

### Mutant construction and complementation studies

To generate non-polar mutations in the 17 *xrp* genes, two fragments flanking each gene were PCR-amplified from *Xoc* RS105 genomic DNA with primer pairs *xrpX*I-F/*xrpX*I-R and *xrpX*II-F/*xrpX*II-R (X refers to the 17 *xrp* genes; 1 to 17, Table S2 in File S1). After verification by sequence analysis, the two fragments flanking each gene were fused and ligated to the suicide vector pKMS1 [57] at selected restriction sites (Table S2 in File S1), producing plasmids pKMSΔ*xrpX* (X refers 1 to 17, Table S1 in File S1). Mutations in the 17 *xrp* genes were generated by two-step homologous recombination as described previously [57]. Deletions in the *xrp* genes were confirmed by PCR-amplification with the primer pairs, *xrpX*I-F/*xrpX*II-R (Table S2 in File S1); the results confirmed that the PCR products were smaller than those obtained in RS105 (data not shown). For complementation studies, constructs designated pXrpXMyc (X refers 1 to 17, Table S1 in File S1) were introduced into the corresponding *xrp* mutants by biparental conjugation. Mutants containing *xrp* genes *in trans* were verified by colony-PCR and named CRΔ*xrp*X (X refers 1 to 17, Table S1 in File S1).

### Bacterial virulence and growth *in planta*

Rice cultivar Shanyou 63 (two weeks old) was used to evaluate the virulence of *Xoc* RS105 and derivatives. Bacterial cells were adjusted to 1×10^8^ cfu/ml and infiltrated into newly-expanded leaves with a needleless syringe at three locations per leaf. Three leaf discs (0.5 cm in diameter) were excised with a cork borer from each infiltrated area. After sterilization in 70% ethanol and 30% hypochlorite, the discs were macerated using a sterile mortar and pestle in 1 ml of distilled water, diluted and plated to determine cfu/cm^2^. Serial dilutions were spotted in triplicate on NA plates with appropriate antibiotics. The plates were incubated at 28°C for 3–4 days until single colonies could be counted. The bacterial population (cfu/cm^2^ of leaf area) was then estimated, and the standard deviation was calculated using colony counts from three triplicate spots of three samples obtained at each time point. All rice cultivars were grown in a greenhouse maintained at 25°C with a 12-h photoperiod. Experiments were repeated at least three times.

Near-isogenic lines of rice cultivar IRBB10 were used to assay the pathogenicity of *Xoo* PXO99^A^ and derivatives as described previously [47]. Plant responses were scored 14 dpi for lesion lengths. Experiments were repeated at least three times.

### Type III secretion assays

The pXrpXMyc plasmids were transformed into the wild-type RS105 and RΔ*hrcV* for detection of secreted proteins. *Xoc* strains were preincubated in NB medium, washed twice, and suspended at OD_600_ = 2.0 with sterilized water. An aliquot (40 µl) of the bacterial suspension was inoculated into 1 ml of XOM3 medium (pH 6.0), adjusted to OD_600_ = 1.0, and incubated at 28°C for 6 h with the appropriate antibiotics. Supernatant fractions were separated using a 0.22 µm filter, and the supernatant fraction (50 ml) was reduced to 5 ml by vacuum evaporation. Proteins were precipitated with 12.5% trichloroacetic acid at 4°C for 16 h, centrifuged at 3000× g for 15 min, and then washed briefly with acetone and air-dried. The protein pellet was resuspended in SDS buffer containing dithiothreitol (DTT) [58]. Proteins were separated on 10% SDS-PAGE gels and transferred to membranes for immunoblotting using primary antibody anti-c-Myc (Huaan, Hangzhou, China). Primary antibodies were recognized by anti-rabbit secondary antibodies (Huaan) and visualized by autoradiography with the Western-Light chemiluminescence system (Transgene, Beijing, China). Experiments were repeated at least twice and Hpa1 was used as a positive control [59].

## Results

### Screening of T3SE candidates from *Xoc*

Genome-wide identification of bacterial virulence genes has been greatly facilitated by the availability of microarrays [60], [61]. Considering the critical fact that HrpG and HrpX are two key regulators for pathogenicity determinants in Xanthomonads [14], [26], [28], [29], [43], [44], the expression profiles of genome widely annotated hypothetical proteins in *Xoc* RS105 and *hrpG* and *hrpX* mutants were only evaluated based on the annotated sequence of *Xoc* BLS256 (AAQN01000001.1 (GI:94721269)). The three *Xoc* strains (RS105, RΔ*hrpG*, RΔ*hrpX*) were co-cultivated with rice cells for 16 h at 25°C, and bacterial RNAs were extracted and hybridized with the BLS256 genechip. The expression levels of hypothetical protein genes in the *hrpG* and *hrpX* mutants were compared with the wild-type RS105 and taken as the candidates if the ratio was lower than 0.55 or higher than 1.75 with the *P* value less than 0.05 (Table S3 in File S1). For a comparison, we checked the expression of *hrp* genes and known T3SE genes (Table S3 in File S1) and found that the expression patterns of these genes *in silicon* were consistent with those that were experimentally explored previously [12], [23], [44], [56], [61] Totally, there were 247 hypothetical protein genes significantly (*P*≤0.05, *t* test) altered their expression levels in the *hrpG* or/and *hrpX* mutants, comparing to the wild-type RS105 (Table S3 in File S1), indicating that these *in silicon data* are helpful to find HrpG/HrpX-regulated proteins (Xrp).

To reveal whether there is(are) any T3SE protein(s) in these Xrp candidates, three criteria are considered: (i) candidate genes are homologous to known T3SEs in other phytopathogenic bacteria [16], [22]; (ii) expression of candidate genes is HrpX-dependent with the presence of a PIP-box in the promoter [29], [38], and (iii) the candidate protein product contains targeting signals at the N-termini that are indicative of T3S secretion [22], [47]. For these, sequences in the *xrp* promoter regions and in the N-termini of Xrp translational products (identical correspondingly to these in *Xoc* BLS256 after sequencing confirmation) were analyzed and the protein IDs were changed into Table 1 and Table 2 according to the new version of the genome sequence of *Xoc* BLS256 (NZ_AAQN01000001.1, GI:353459993). In Xrp1, Xrp3, Xrp4,,Xrp5, Xrp7, Xrp8, Xrp9, Xrp11 and Xrp14, the total number of Ser and Pro residues in the first 50 N-terminal amino acids constitutes more than or equal to eight (Table 1). Xrp1, Xrp3, Xrp4, Xrp5, Xrp6, Xrp8 and Xrp11 contain at least five Ser residues at the N-terminus (Table 1). Promoter analysis showed that there are PIP-box-like sequences upstream of *xrp1*, *xrp2*, *xrp8*, *xrp9*, *xrp13*, *xrp14*, *xrp15* and *xrp16* ORFs (Table 1). Interestingly, these PIP-box-like sequences, which contained 9–25 nucleotides between TTCGB motifs, differ from the typical consensus PIP-box (TTCGB-N_15_-TTCGB). To have a better understanding, three candidates (Xrp10, Xrp12 and Xrp17) without either of two properties abovementioned were selected from 247 hypothetical protein genes (Table S3 in File S1) as the comparison.

**Table 1:** Protein and nucleotide sequence analysis of 17 *xrp* genes.

RS105 *Xrp*	Protein ID in BLS256	Gene ID in BLS256	Ser and Pro^a^	Ser	Pro	Leu^b^	Asp and Glu^c^	3^rd^ AA	4^th^ AA	PIP box-like^d^		−10 Box-like
										position	5′-3′ sequence	
Xrp1	YP_005627937	XOC_1601	8	6	2	8	0	T	H	−96/−75	TTCGC-N_12_-TTCGC	N^e^
Xrp2	YP_005630809	XOC_4584	6	4	2	2	0	K	F	−106/−85	TTCGT-N_12_-TTCGT	N
Xrp3	YP_005630213	XOC_3956	10	6	4	3	1	T	R		N	N
Xrp4	YP_005630212	XOC_3955	8	6	2	7	2	R	H		N	N
Xrp5	YP_005627898	XOC_1550	8	7	1	7	2	G	E		N	N
Xrp6	YP_005629702	XOC_3440	6	5	1	4	1	V	E		N	N
Xrp7	YP_005630808	XOC_4583	9	2	7	6	1	S	L		N	N
Xrp8	YP_005628761	XOC_2462	9	6	3	6	1	G	L	−81/−56	TTCGA-N_16_-TTCGA	N
Xrp9	YP_005628272	XOC_1951	11	3	8	8	0	L	R	−114/−69	TTCGT-N_6_-TTCGA-N_25_-TTCGC	-N33-TATGAT
Xrp10	YP_005626937	XOC_0560	6	2	4	1	0	V	P		N	N
Xrp11	YP_005629396	XOC_3130	8	8	0	4	1	I	Q		N	N
Xrp12	YP_005630266	XOC_4010	5	2	3	8	1	A	L		N	N
Xrp13	YP_005627233	XOC_0860	5	1	4	5	1	L	A	−240/−211	TTCGC-N_20_-TTCGG	N
Xrp14	YP_005626509	XOC_0084	10	3	7	4	2	D	D	−61/−26	TTCGC-N_25_-TTCGC	N
Xrp15	YP_005629104	XOC_2829	2	1	1	5	1	I	E	−121/−90	TTCGA-N_21_-TTCGC	N
Xrp16	YP_005629103	XOC_2828	5	3	2	5	0	A	P	−362/−331	TTCGA-N_22_-TTCGG	N
Xrp17		*hrpFB*	2	2	0	6	1	Y	F		N	N

a Number of Ser and Pro residues in the N-terminal 50 amino acids.

b Number of Leu residues in the N-terminal 50 amino acids.

c Number of Asp and Glu residues in the N-terminal 12 amino acids.

d Includes an imperfect PIP-box like sequence (TTCGB-Nx-TTCGB,B,A/T/C/G) in the respective promoter.

e No corresponding characteristics.

**Table 2:** Conservation of 17 Xrps of *X. oryzae* pv. *oryzicola* RS105 in other *Xanthomonas* species.

*Xoc* RS105	Description	*Xoc* BLS256	*Xoo*			*Xac* 306	*Xcv* 85-10	*Xcc* ATCC33913
			PXO99^A^	MAFF311018	KACC10331			
Xrp1	cysteine protease	XOC_1601 (100%)^a^	PXO_04730 (83%)	XOO1385 (84%)	XOO1487 (83%)	XAC2853 (88%)	XCV3013 (87%)	XCC2693 (83%)
Xrp2	hypothetical protein	XOC_4584 (100%)	PXO_03859 (82%)	XOO4169 (82%)	XOO4426 (82%)	N	XCV0093 (54%)	N
Xrp3	hypothetical protein	XOC_3956 (100%)	PXO_03076 (88%)	XOO0633 (89%)	XOO0696 (89%)	XAC3685 (88%)	XCV3806 (86%)	XCC3645 (79%)
Xrp4	hypothetical protein	XOC_3955 (100%)	PXO_03077 (85%)	XOO0634 (85%)	XOO0697 (85%)	XAC3684 (78%)	XCV3805 (80%)	XCC3644 (60%)
Xrp5	hypothetical protein	XOC_1550 (100%)	PXO_04764 (89%)	XOO1359(89%)	XOO1457 (89%)	XAC2878 (81%)	XCV3033 (82%)	XCC2715 (80.8%)
Xrp6	hypothetical protein	XOC_3440 (100%)^b^	PXO_01952 (93%)	N	N	N	N	N
Xrp7	glucuronate isomerase	XOC_4583 (100%)	PXO_03860 (96%)	XOO4170 (96%)	XOO4427 (95%)	XAC4251 (92%)	XCV4357 (92%)	XCC4117 (86%)
Xrp8	hypothetical protein	XOC_2462 (100%)	N^a^	N	N	XAC2155 (91%)	XCV2099 (91%)	XCC2020 (86%)
Xrp9	hypothetical protein	XOC_1951 (100%)	PXO_00529 (98%)	XOO2357 (99%)	XOO2488 (96%)	XAC2517 (96%)	XCV2699 (92%)	XCC2382 (84%)
Xrp10	hypothetical protein	XOC_0560 100%)	PXO_04113 (90%)	XOO3892 (91%)	XOO4113 (90%)	XAC3866 (94%)	XCV3985 (94%)	XCC3811 (81%)
Xrp11	hypothetical protein	XOC_3130 (100%)	PXO_01766 (85%)	XOO1798 (85%)	XOO1902 (85%)	XAC1364 (85%)	XCV1420 (85%)	XCC1318 (83%)
Xrp12	S-(hydroxymethyl) glutathione dehydrogenase	XOC_4010 (100%)	N	XOO0585 (96%)	XOO0635 (95%)	XAC3747 (92%)	XCV3866 (94%)	XCC3703 (90%)
Xrp13	Protease	XOC_0860 (100%)	PXO_04349 (96%)	XOO3588 (97%)	XOO3806 (96%)	XAC0795 (79%)	XCV0845 (80%)	N
Xrp14	cytochrome P450 BJ-1	XOC_0084 (100%)	N	N	N	XAC3170 (29%)	N	N
Xrp15	NDP-hexose isomerase	XOC_2829 (100%)	PXO_00207 (98%)	XOO2849 (96%)	XOO2998 (98%)	XAC1687 (92%)	XCV1723 (93%)	XCC1670 (86%)
Xrp16	transferase hexapeptide repeat	XOC_2828 (100%)	PXO_00206 (94%)	XOO2848 (94%)	XOO2997 (94%)	XAC1688 (86%)	XCV2006 (33%)	N
Xrp17	hypothetical protein	HrpFB^c^ (100%)	N	N	N	N	N	N

a Parentheses indicate percent amino acid identity. N, no homologous gene was identified.

b XOC_3440,an ORF located between *hrpG* and *hrpX*.

c HrpFB, an ORF located upstream of *hrpF*.

**Figure 1:**
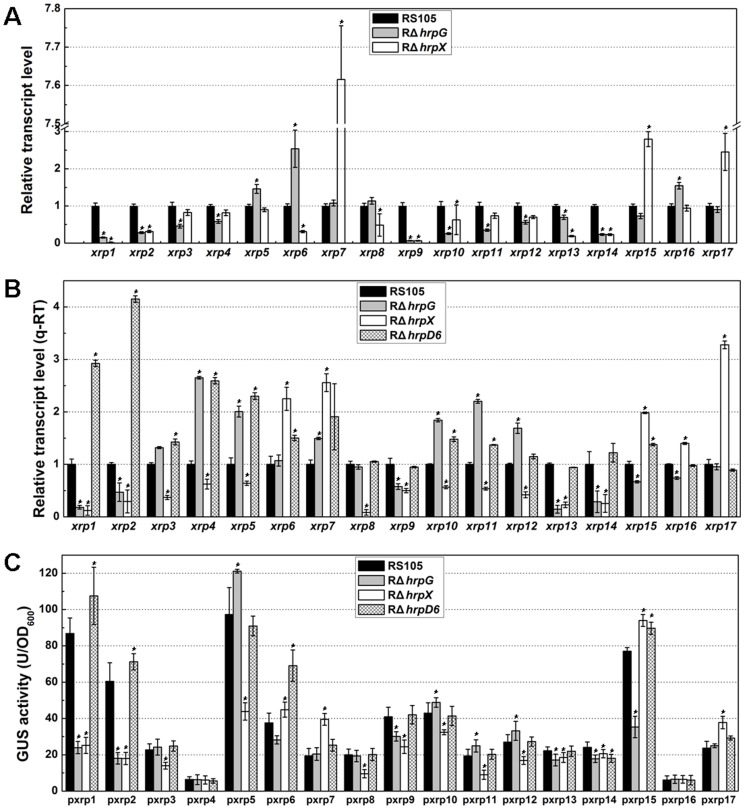
Transcriptional expression of 17 *X. oryzae* pv. *oryzicola xrp* genes exposed to three *hrp*-inducing conditions. (A) The transcriptional profiles *in silicon* of 17 *xrp* genes in *Xoc* RS105, RΔ*hrpG* and RΔ*hrpX*. The three strains were incubated with rice cells at 28°C for 16 h, and RNA was extracted and hybridized to the genechip arrays described in Materials and Methods. (B) Expression of 17 *xrp* genes using qRT-PCR. RNAs were extracted from *Xoc* RS105, RΔ*hrpG*, RΔ*hrpX* and the *hrpD6* mutant (RΔ*hrpD6*) and used to synthesize cDNA for qRT-PCR. *gyrB* and *rpoD* were used as internal controls. (C) GUS activities of 17 *xrp* promoter-GUS fusions in strains RS105, RΔ*hrpG*, RΔ*hrpX* and RΔ*hrpD6*. Strains were cultured in XOM3 at 28°C for 6 h, and GUS activities were determined by measuring using 4-MUG as a substrate. Data are the mean ± SD of triplicate measurements from a representative experiment; and similar results were obtained in two other independent experiments. Asterisks in horizontal columns indicate significant differences using the Student's *t* test (*P<0.05).

The *in silicon* data showed that the expression of *xrp1*, *xrp2*, *xrp9*, *xrp13*, *xrp14* was significantly (*t* test, *P*≤0.05) positively regulated by both HrpG and HrpX. the expression of *xrp8* was positively regulated only by HrpX, the expression of *xrp3*, *xrp4*, *xrp11* and *xrp12* was positively regulated only by HrpG, the expression of *xrp7 xrp15*, and *xrp17* was negatively regulated by HrpX, and the expression of *xrp5*, *xrp6* and *xrp16* was significantly negative regulated only by HrpG with respect to expression levels in the wild-type RS105 (Fig. 1A, Table S3 in File S1). These results suggest that mutations in *hrpG* or *hrpX* may alter the expression of these 17 *xrp* genes during bacterial infection in rice.

### Expression patterns of *xrp* genes controlled by HrpG, HrpX and HrpD6

To confirm the expression profiles of these 17 *xrp* genes in RS105, RΔ*hrpG*, and RΔ*hrpX* (Fig. 1A), we used quantitative real-time PCR (q-RT PCR) to determine whether the expression was HrpG or HrpX-dependent. Primers (Table S2 in File S1) selected for this experiment were based on the genome of BLS256 (NZ_AAQN01000001.1, GI:353459993)(10). Since HrpD6 is a newly identified *hrp* regulatory factor, we also investigated whether the expression of these 17 *xrp* genes was altered in the mutant RΔ*hrpD6*. Controls included Hpa1, which contains a T3S signal at the N-terminus and the PIP-box promoter [24], [59] and XOC_0618, which is homologous to XopX [22]. Hpa1 and XOC_0618 were positively regulated by HrpG and HrpX, but negatively regulated by HrpD6 (Fig. S1 in File S1); this observation is consistent with previous results [12]. As shown in Fig. 1B, the expression of *xrp1*, *xrp2*, *xrp9*, *xrp13* and *xrp14* was significantly positively regulated by both HrpG and HrpX, which is consistent with our *in silicon* data (Fig. 1A, Table S3 in File S1); the expression of *xrp3* and *xrp8* was decreased in the *hrpX* mutant, relative to the wild-type and *hrpG* mutant; the expression of *xrp4*, *xrp5*, *xrp10*, *xrp11* and *xrp12* was decreased in RΔ*hrpX*, but increased not only in RΔ*hrpG* but also in RΔ*hrpD6*; and the expression of *xrp6*, *xrp7*, *xrp15 xrp16* and *xrp17* was significantly increased in the *hrpX* mutant. The qRT-PCR results (Fig. 1A) were generally consistent with the expression *in silicon* (Fig. 1). In contrast, the expression of *xrp1*, *xrp2*, *xrp3*, *xrp4*, *xrp5*, *xrp6*, *xrp7*, *xrp10*, *xrp11*, and *xrp15* was significantly increased in the *hrpD6* mutant (Fig. 1B).

To further investigate the expression of these 17 *xrp* genes, we fused the putative promoter regions (500 bp upstream of the translational start site) to a promoterless *gusA* gene. The promoters were PCR-amplified from genomic DNA of strain RS105 using the primers listed in Table S2 in File S1. After sequencing these PCR products from RS105, no obvious difference was found in corresponding regions in BLS256 genome (data not shown). The pxrp-*gusA* fusions were then transferred into the wild-type RS105, the *hrpG*, *hrpX* and *hrpD6* mutants, respectively, then incubated in XOM3 (a *hrp*-inducing medium) [49] at 28°C for 6 h. The GUS expression patterns (Fig. 1C) detected by these 17 *xrp* promoters was similar to the regulation observed by genechip and qRT-PCR analysis (Figs. 1A, B). However, there were no obvious differences in GUS activities expressed from the *xrp4*, *xrp13*, *xrp14* and *xrp16* putative promoters in RS105, and the *hrpG*, *hrpX* and *hrpD6* mutants (Fig. 1C), possibly because that these four genes localize within adjacent operons where the tested promoters are not real for GUS activity detection (data not shown).

### *xrp1*, *xrp2*, *xrp5*, *xrp8* and *xrp14* genes are required for *Xoc* virulence to rice

The expression patterns of these 17 *xrp* genes in the *hrpG*, *hrpX* and *hrpD6* mutant backgrounds prompted us to determine whether they are involved in *Xoc* virulence. Each *xrp* gene was PCR-amplified using primers derived from the BLS256 genome (Table S2 in File S1). Sequence and BLAST analysis of *xrp* PCR products from RS105 showed no differences in these *xrp* genes with the corresponding ORFs in BLS256 (Table 2). All these Xrp proteins of RS105 showed 100% identity to the homologs of BLS256 (Table 2). Homologs of Xrp2 were not identified in *X. axonopodis* pv. *citri* strain 306 (*Xac* 306) and *X. campestris* pv. *campestris* (*Xcc*) ATCC33913. Xrp6 was only identified in *Xoc* BLS256 and *Xoo* PXO99^A^; Xrp8 had no orthologs in *Xoo* strains but in other three *Xanthomonas* species, and Xrp12 was not present in *Xoo* PXO99^A^. Xrp14 was identified in *Xoc* BLS256, but only had lower similarity (29%) in *Xac* 306; Xrp13 and Xrp15 had no homologous counterparts in *Xcc*; and Xrp17 was a putative ORF upstream of HrpF only in *Xoc* BLS256 genome (designed HrpFB herein) (Table 2). It should be mentioned here that the ORFs and putative products for *xrp1*, *xrp7*, *xrp12*, *xrp13*, *xrp14*, *xrp15* and *xrp16* were re-annotated in the new version of *Xoc* BLS256 genome sequence (NZ_AAQN01000001.1, GI:353459993) (Table 2).

**Figure 2:**
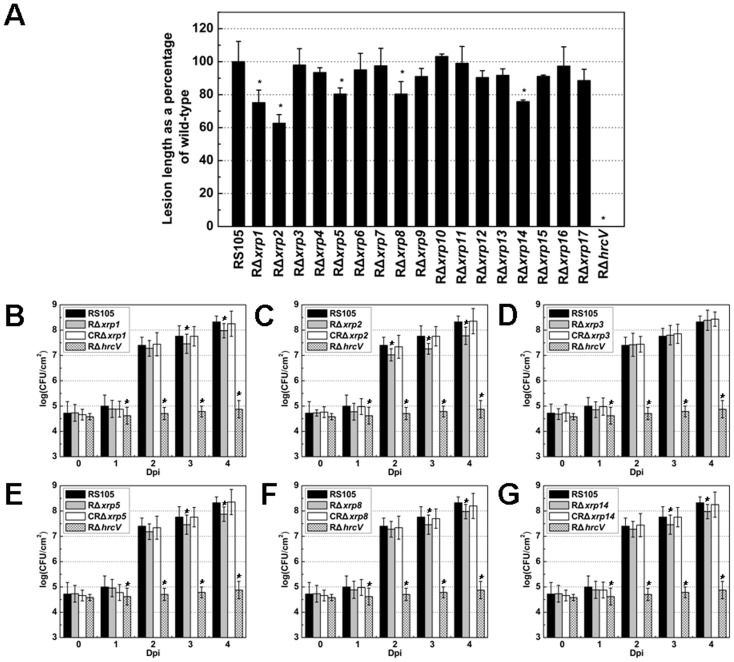
Virulence evaluation of 17 *xrp* mutants of *X. oryzae* pv. *oryzicola* in rice. (A) Lesion lengths of 17 *xrp* mutants derived from the wild-type strain RS105. Lesion lengths were measured as the ratio of lesion length caused by an *xrp* mutant compared to the wild-type RS105. The *hrcV* mutant was used as a negative control. *Xoc* strains (∼1×10^8^ cfu/mL) were inoculated to rice cv. Shanyou 63 (two-months old) by leaf-needling [58]. Lesion lengths were scored 14 days post inoculation. Data are the mean ± standard deviation (SD) of three replicates, and the data shown are representative of three independent experiments. Asterisks at the top of columns indicate significant differences by the Student's *t* test (*P<0.05). Panels B through G show the population dynamics of *Xoc* RS105 and derivatives in rice leaves. Leaf discs (0.5 cm in diameter) were excised from the inoculated areas, homogenized in sterile water, diluted and plated on nutrient agar (NA) plates. Panels: (B), *xrp1* mutant, RΔ*xrp1*; (C), *xrp2* mutant, RΔ*xrp2*; (D), *xrp3* mutant, RΔ*xrp3*; (E), *xrp5* mutant, RΔ*xrp5*; (F), *xrp8* mutant, RΔ*xrp8*; and (G) *xrp14* mutant, RΔ*xrp14*. Similar results were obtained in two other independent experiments.

In order to explore the putative role of the *xrp* genes in virulence, we generated in-frame deletion mutants of the 17 *xrp* genes (Table S1 in File S1) in *Xoc* RS105 as described previously [57]. The left and right fragments flanking the *xrp* genes were PCR-amplified with primers listed in Table S2 in File S1 using RS105 genomic DNA as the template, fused, transferred into the suicide vector pKMS1 and generated deletion mutants correspondingly (Table S1 in File S1). These 17 nonpolar deletion mutants were inoculated into leaves of rice cv. Shanyou 63, which is susceptible to *Xoc*. The lesion lengths generated by *xrp1*, *xrp2*, *xrp5*, *xrp8* and *xrp14* mutants were significantly shorter than those produced by *Xoc* RS105 (Fig. 2A), and these five mutants were impaired in their ability to grow in rice leaves compared to the wild-type (Fig. 2B–G). Virulence and bacterial growth *in planta* were restored to wild-type levels by the introduction of corresponding *xrp* genes *in trans* (Fig. 2B–G). The above data indicate that *xrp1*, *xrp2*, *xrp5*, *xrp8* and *xrp14* genes are required by *Xoc* for bacterial virulence and growth in rice.

### Xrp3 and Xrp5 are novel T3SEs

**Figure 3:**
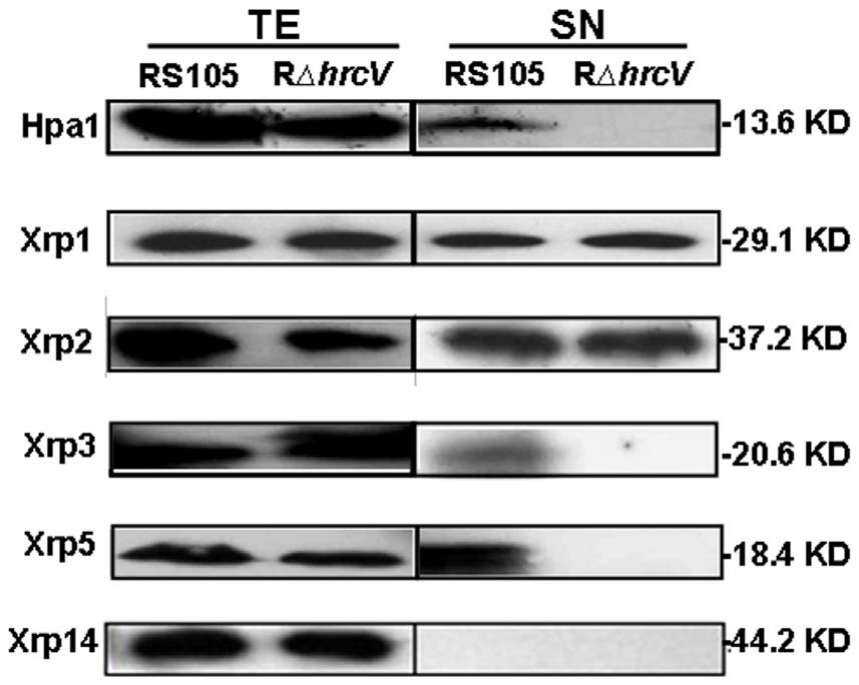
Secretion detection of c-Myc epitope-tagged derivatives of Xrp proteins through the T3S. *Xoc* wild-type strain RS105 and the T3S mutant RΔ*hrcV*, harboring c-Myc eptope-tagged Xrp protein constructs were incubated in a *hrp*-inducing medium XOM3. Total protein extracts (TE) and culture supernatants (SN) of the tested strains were analyzed by SDS-PAGE and immunoblotted using anti-c-Myc antibodies. Immunoblots were performed described in Materials and Methods. Hpa1 was used as a positive control. Similar results were obtained in two other independent experiments.

In the complementation studies mentioned above, a c-Myc tag was fused in frame at the C-termini of the 17 Xrp proteins (see Table S1 in File S1), which facilitated subsequent expression studies. The c-Myc-tagged constructs were introduced into *Xoc* RS105 and RΔ*hrcV* [62] and incubated in *hrp*-inducing medium XOM3 [48] for 6 h. The supernatants (SN) and total extracts (TE) of bacterial cells were used to investigate whether the Xrps were expressed by Western blotting using a c-Myc specific polyclonal antibody (Huaan, Hangzhou, China). With the exception of Xrp4, Xrp6, Xrp9, Xrp10 and Xrp13, the other 12 Xrp proteins were detectable in TEs of both RS105 and RΔ*hrcV* (representative data are shown in Fig. 3). However, Xrp3 and Xrp5, like Hpa1, were detected in SNs of the wild-type, but not in the T3S mutant (Fig. 3). Xrp1 and Xrp2 were detected in the SN fractions of both the wild-type and the T3S mutant, whereas Xrp14 were not (Fig. 3). These data indicate that Xrp3 and Xrp5 are secreted via the T3S, but Xrp1 and Xrp2 may be secreted via other systems.

**Figure 4:**
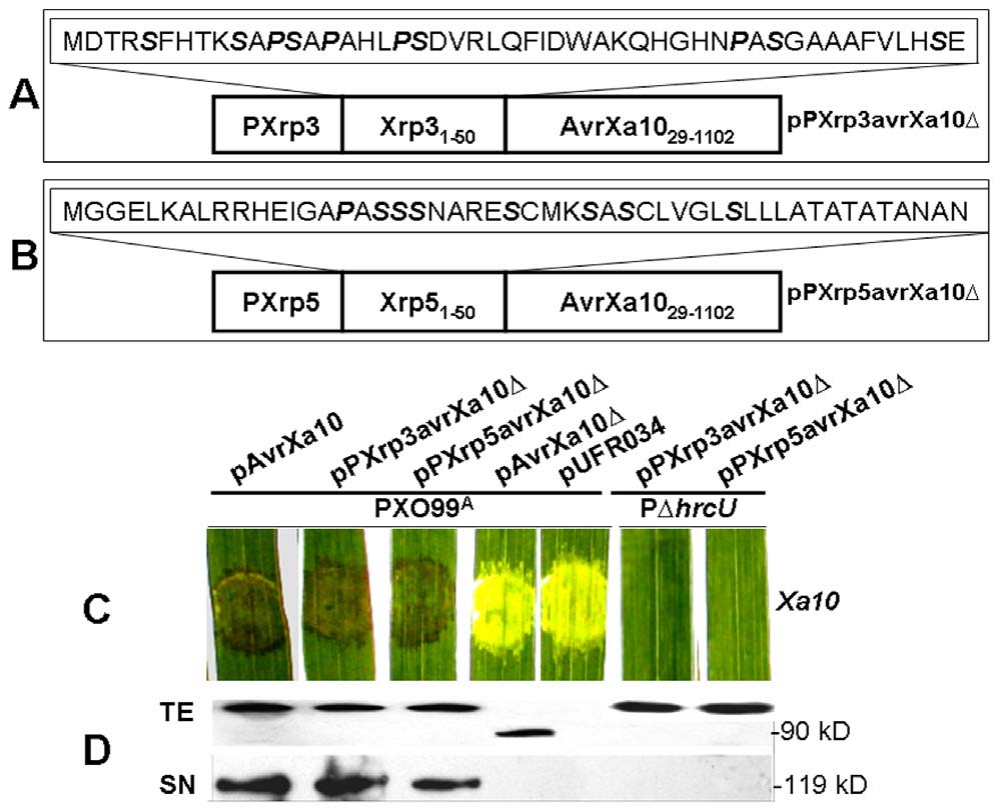
Translocation of Xrp3 and Xrp5 into plant cells via the T3S. Panels (A) and (B) show schematic maps of fusion proteins Xrp3 and Xrp5, respectively. The putative promoter and 5′ coding regions of *xrp3* and *xrp5* were fused to AvrXa10Δ28; this resulted in constructs containing in-frame fusions between with the N-terminus of Xrp3/Xrp5 and truncated AvrXa10Δ28 (AvrXa10_29-1211_). The 50 N-terminal amino acids in Xrp3 and Xrp5 are indicated in dashed boxes. (C) Avirulence activities of pXrp3avrXa10Δ28 and pXrp5avrXa10Δ28 in leaves of rice IRBB10 carrying the resistance gene *Xa10*. The secondary leaves of 14-day-old seedlings of IRBB10 were inoculated by needleless syringe with the respective bacterial strains as illustrated. Photos were taken 3 days after inoculation. The procedure was performed as in Materials and Methods, and similar results were obtained in two other independent experiments. (D) Detection of Xrp3 and Xrp5 secretion via the T3S in *Xoc* strains by immunoblotting. PXO99^A^ and RΔ*hrcU* containing c-Myc tagged derivatives of pXrp3avrXa10Δ28 and pXrp5avrXa10Δ28 were incubated in secretion medium XOM3. Total protein extracts (TE) and culture supernatants (SN) were analyzed by SDS-PAGE and immunoblotted using anti-c-Myc antibodies. The experiment was performed as described in Materials and Methods, and similar results were obtained in two other independent experiments.

*Xanthomonas* T3SEs containing up to 20% Ser/Pro residues or at least five Ser residues in their N-terminal sequences are deemed to contain a T3S signal [22], [33]. One strategy for determination of T3SE secretion via the T3S is to fuse the N-terminus of a candidate effector with an Avr protein that lacks the secretion signal, and investigate whether the fused effector can be translocated into plant cells [24], [38]. The first 50 N-terminal amino acid residues of Xrp3 and Xrp5 contain a total of 10 and 8 Ser/Pro residues, respectively (Fig. 4A, B), indicating that Xrp3 and Xrp5 may be translocated into host cells. To further investigate this possibility, the N-terminal 50 amino acids of Xrp3 and Xrp5 were fused with a truncated AvrXa10 that lacked 28 amino acid residues at the N-terminus (avrXa10Δ). The chimeric proteins Xrp3avrXa10Δ and Xrp5avrXa10Δ were constructed using native *xrp* promoters (see Materials and Methods), resulting in pPXrp3avrXa10Δ and pPXrp5avrXa10Δ, respectively (Table S1 in File S1, Fig. 4A, B). The wild-type RS105 harbouring *avrXa10* does not trigger an HR in rice line IRBB10, which carries the cognate *R* gene *Xa10* [24]; thus, we utilized *Xoo* strain PXO99^A^ to investigate whether the fused proteins can trigger an HR in IRBB10. The T3S mutant PΔ*hrcU* was used as a negative control. PXO99^A^ containing pPXrp3avrXa10Δ or pPXrp5avrXa10Δ produced an HR in IRBB10, as did PXO99^A^ (pavrXa10) (Fig. 4C). However, HR was not elicited by PXO99^A^ containing pavrXa10Δ or the empty vector pUFR034 (Fig. 4C). As predicted, the T3S mutant PΔ*hrcU* did not elicit symptoms in cultivar IRBB10 (Fig. 4C). We then used immunoblotting to determine whether the translational fusions present in pPXrp3avrXa10Δ and pPXrp5avrXa10Δ were expressed in the tested strains. As expected, the chimeric proteins PXrp3avrXa10Δ and PXrp5avrXa10, like the wild-type AvrXa10 and the N-terminal truncated AvrXa10Δ, were detectable in the TEs of both wild-type PXO99^A^ and PΔ*hrcU*, but not in SN fraction of PΔ*hrcU* (Fig. 4D). The above data indicate that the N-terminal portions of Xrp3 and Xrp5 enable the N-terminal truncated AvrXa10 to be secreted through the T3S and translocated into rice cells for HR induction.

### Subcellular localization of Xrp3 and Xrp5

**Figure 5:**
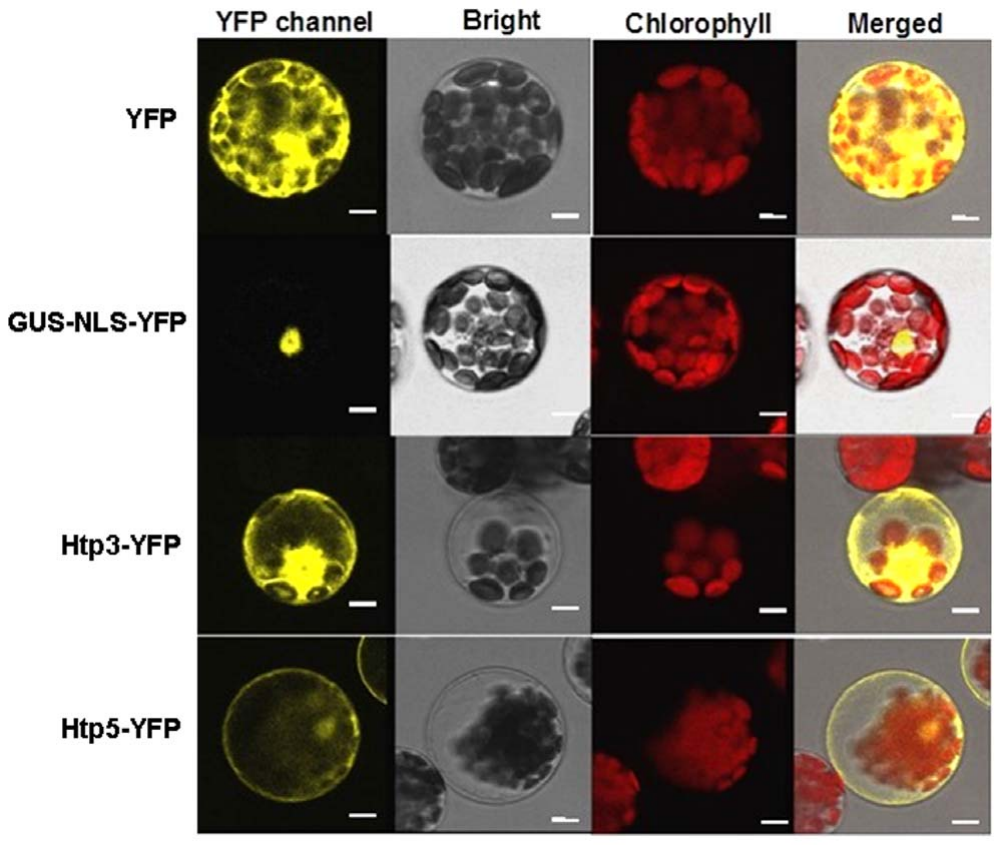
Subcellular localization of Xrp3 and Xrp5 in plant cells. The subcellular localization of Xrp3 and Xrp5 was examined by fusing these proteins with yellow fluorescence protein (YFP) and evaluating their expression in *Arabidopsis* mesophyll cells. Images were generated by fluorescence at 525–550 nm (YFP, yellow) and 610–700 nm (chloroplasts, red) using excitation at 514 nm. Expression was driven by the CaMV 35S promoter. PEG-calcium-mediated transfection was used to deliver DNA into protoplasts, and photos were taken 12 h after transformation. Controls included YFP, which localizes in both cytoplasm and nucleus, and GUS-NLS-YFP, which localizes in the nucleus. Bars correspond to 10 µm. The experiment procedure was performed as described previously [54] and repeated three times with similar results.

The localization of Xrp3 and Xrp5 was examined by utilizing YFP-tagged Xrp3 and Xrp5 proteins. The protocol (see Materials and Methods) utilizes mesophyll protoplasts of *Arabidopsis* and PEG-calcium-mediated transfection to deliver pXrp3-YFP and pXrp5-YFP (Table S1 in File S1) into plant cells; GUS-NLS-YFP and YFP were used as a reference. Fluorescence confocal microscopy indicated that YFP-tagged Xrp3 proteins are localized throughout the cell, but Xrp5-YFP is targeted to the plasma membrane and cytoplasm. The latter result was clearly different from the YFP control, which was partially retained in the nucleus (Fig. 5). In general, our results suggest that Xrp3-YFP is localized throughout the cell (cytoplasm, nucleus, plasma membrane) and Xrp5-YFP preferentially targets the cytoplasm.

## Discussion

In this report, two new T3SEs, Xrp3 (XOC_3956) and Xrp5 (XOC_1550), were identified and shown to be HrpX-dependent based on transcriptional data *in silicon* (Fig. 1A). These two newly-identified T3SEs can be added to the extensive list of *Xanthomonas* T3SEs at http://www.Xanthomonas.org/t3e.html [16]. Interestingly, *xrp3* and *xrp5* are highly conserved in Xanthomonads rather than other plant pathogenic bacteria whose genomes have been completely sequenced (Table 2). Xrp5, but not Xrp3, was important for a full level of virulence in rice (Fig. 2). This is consistent with a previous study that many NTALEs but XopZ in *Xoo* do not impact bacterial virulence in rice [63].

The *in silicon* and qRT-PCR data (Figs. 1A, B) consistently showed that the expression of 17 *xrp* genes was regulated by HrpX and/or HrpG at the transcriptional level. However, GUS activity detection showed that the expression of *xrp4* (*XOC_3955*) and *xrp16* (*XOC_2828*) did not display any obvious differences in regulation in WT and mutant strains (Fig. 1C), being due to that they are members of their adjacent operons (data not shown). Giving that the expression of *xrp1* (*XOC_1601*), *xrp2* (*XOC_4584*), *xrp3* (*XOC_395*6), *xrp4* (*XOC_3955*), *xrp5* (*XOC_1550*), *xrp6* (*XOC_3440*), *xrp7* (*XOC_4583*), *xrp10* (*XOC_0560*), *xrp11* (*XOC_3130*) and *xrp15* (*XOC_2829*) was negatively regulated by HrpD6 but either positively regulated by HrpX and/or HrpG or negatively regulated by HrpX (Fig. 1B), HrpG-, HrpX- and HrpD6-mediated regulation in xanthomonads is very complicated to follow the concept that *hrpX* expression is controlled by HrpG [14], [44] and HrpX regulates the expression of *hrpD6* [12]. In general, one criterion for identification of HrpX regulon genes is the PIP-box [26], [27], [43], [64]. However, PIP-box-like sequences are absent in the *xrp3* and *xrp5* upstream regions (Table 1). This is consistent with reports that the expression of some HrpX-regulated genes that lack PIP-box promoters is modulated directly by HrpX and indirectly by HrpG [22]. For example, there is no PIP-box in the promoter regions of *hpaJ*, *XCV_0869* or *XCV_3406* genes, but their expression is HrpX-dependent [26]. Thus, there may be an alternative regulatory system in *Xanthomonas* where HrpX activates genes independently via an unknown regulator(s), like HrpD6.

It should be emphasized that the location of *xrp6* (*XOC_3440*) and *xrp17* (*hrpFB*) is within the *hrp-hrc-hpa* cluster. *xrp6* is a putative ORF between *hrpG* and *hrpX*, and *xrp17* is *hrpFB* upstream of *hrpF*. These two ORFs have not been annotated in other *Xanthomonas* genomes (Table 1). Our data suggest that *xrp6* and *xrp17* are not involved in *Xoc* virulence (Fig. 2A); however, the expression of these two ORFs was negatively regulated by HrpX, but not affected by HrpG (Fig. 1B), and the gene product of *xrp17* was not detectable in the SN of the WT and mutant strains (data not shown). It remains unclear whether Xrp6 or Xrp17 function in the regulation of the *hrp-hrc-hpa* cluster.

New *Xoc* virulence factors identified in this study included Xrp1 (XOC_1601), Xrp2 (XOC_4584), Xrp8 (XOC_2462), and Xrp14 (XOC_0084) (Fig. 2). Xrp1 (XOC_1601) encodes a putative cysteine protease, which is highly conserved in *Xoo*, *Xac*, *Xcc*, and *Xcv* (Table 2); this protein shows 84% identity with the T2SS protein CysP2 in *Xoo* [14], XOC_1601 is controlled by DSF-mediating QS (quorum-sensing) in *Xoc* RS105, involved in extracellular protease activity, cell motility, antioxidative ability and EPS biosynthesis [65]. However, Xrp1 is not related to XopD, AvrXv4, AvrPphB or AvrRpt2, which have cysteine protease functions that alter plant immunity [66]–[71]. Given that Xrp1 is involved in *Xoc* virulence in rice (Fig. 2) and T3S-independent secretion (Fig. 3), we assume that this cysteine protein may be secreted via the T2SS, like the homolog of CysP2 in *Xoo* [14]. Xrp1 could potentially degrade a component of the plant cell walls, and the proteolysis of host substrates may be employed by the pathogen to alter plant physiological processes.

Xrp2 (XOC_4584) is conserved in *Xoc*, *Xoo* and *Xcv*, but not in *Xcc* or *Xac* (Table 2); database searches provided no functional clues regarding Xrp2 function. The expression of Xrp2 is HrpG- and HrpX-dependent, but upregulated by HrpD6, like the expression of Xrp1 (Fig. 1). Thus, in addition to regulating *hrp* gene expression [12], HrpD6 may also regulate other virulence factors that are not secreted via the T3S. We found that Xrp8 (XOC_2462) is highly conserved in *Xoc*, *Xcv*, *Xac* and *Xcc*, but has no homolog in *Xoo* strains PXO99^A^, MAFF311018 or KACC10331 (Table 2); thus, this virulence factor (Fig. 2F) may potentially be required for full virulence of *Xcv*, *Xac* and *Xcc* in plants.

Xrp14 (XOC_0084) is present in *Xoc* BLS256 and *Xac* 306 (Table 2) and encodes a putative gene belonging to the cytochrome P450 family [9]. The P450 family proteins use heme iron to oxidize molecules, often making them more water-soluble [72]. It is interesting to note that there are three Ser and seven Pro residues at the N-terminus of Xrp14 (Table 1), indicating that Xrp14 is a potential T3SE. However, the protein is undetectable in the SN of the wild-type strain and T3S mutant (Fig. 3), suggesting that the involvement of Xrp14 in *Xoc* virulence is worthy of further investigation.

In *Xanthomonas* spp., T3SEs have been identified based on sequence similarities identified from genome sequence data [16], [22], avirulence reporter fusion assays [38], Cya-fusion approaches [22] and functional assays of T3S-dependent expression and secretion [73]. It has been demonstrated that the fusion expression of an N-terminally truncated *avr* gene (*avrXa10*) with the first 50 N-terminal amino acids of a T3SE is an important tool for identifying *Xoc* T3SEs by utilizing the *Xoo*-rice pathosystem [24], [47], [74]. However, it may be important to utilize the highly sensitive Cya assay to identify *Xoc* T3SEs [22], [33], [75]. In *R. solanacearum*, 72 T3SEs have been characterized [76]. To date, approximately 26 NTALEs and 29 TALEs have been confirmed or predicted in *Xoc* BLS256 (http://www.xanthomonas.org/t3e.html), excluding HrpE3 [74], and Xrp3 (XOC_3956) and Xrp5 (XOC_1550). Considering the long period of co-evolution of *Xoc* and rice, we speculate that the number of T3SEs in *Xoc* or *Xoo* may possibly be more than the currently identified. Hence, identifying T3SEs is still an important endeavor in *Xoc* where HrpX-regulated proteins could potentially function as T3SEs.

## Supporting Information

File S1 **File containing supporting Figure S1 and Tables S1–S3.**
(DOC)
