# Expression of Concern: Identification of 17 HrpX-Regulated Proteins Including Two Novel Type III Effectors, XOC_3956 and XOC_1550, in *Xanthomonas oryzae* pv. *oryzicola*

**DOI:** 10.1371/journal.pone.0271868

**Published:** 2022-07-18

**Authors:** The PLOS ONE Editors

After this article [1] was published, concerns were raised about the availability of the microarray data generated in this article. The *PLOS ONE* data availability policy [2] applicable at the time the article was submitted to the journal requires authors to make data available upon reasonable request and states that authors must comply with current best practice in their discipline for the sharing of data through databases. As per the 2014 policy [2], best practices in the discipline included the deposition of microarray data.

The authors provide in S2 File a subset of the microarray sequencing data reported in [1]; however, the full dataset is unavailable. In the light of this, the editors issue this Expression of Concern to relay the supporting data provided by authors post-publication and make readers aware of the breach of the journal’s data availability policy.

## Supporting information

S2 File Supplementary dataset. Subset of the microarray sequencing data.
(XLS)
