# Supplementary material for: Identification of 17 HrpX-Regulated Proteins Including Two Novel Type III Effectors, XOC_3956 and XOC_1550, in Xanthomonas oryzae pv. oryzicola
Source: PLoS One. 2014 Mar 27;9(3):e93205. doi: 10.1371/journal.pone.0093205 (PMC3968052; doi:10.1371/journal.pone.0093205)
Supplement: File S1 — File containing supporting Figure S1 and Tables S1–S3. (DOC) [file pone.0093205.s001.doc]

**Identification of 17 HrpX-Regulated Proteins including Two Novel Type III Effectors, XOC_3956 and XOC_1550, in *Xanthomonas oryzae* pv. *oryzicola***

Xiao-bo Xue, Li-fang Zou, Wen-xiu Ma, Zhi-yang Liu, Gong-you Chen

**SUPPORTING INFORMATION**

**Table S1. Strains and plasmids used in this study**

| **Strains or plasmids** | **Relevant characteristics** | **Source** |
| --- | --- | --- |
| ***X. oryzae* pv*. oryzicola*** | | |
| RS105 | Wild-type, causal agent of rice bacterial leaf streak, Rif^r^ | This lab |
| RΔ*hrpG* | *hrpG* deletion mutant of strain RS105; Rif^r^ | This lab |
| RΔ*hrpX* | *hrpX* deletion mutant of RS105; Rif^r^ | This lab |
| RΔ*hrpD6* | *hrpD6* deletion mutant of RS105; Rif^r^ | This lab |
| RΔ*hrcV* | *hrcV* deletion mutant of RS105; Rif^r^ | This lab |
| RΔxrp1 | *XOC_1601* deletion mutant of RS105; Rif^r^ | This study |
| RΔxrp2 | *XOC_4584* deletion mutant of RS105; Rif^r^ | This study |
| RΔxrp3 | *XOC_3956* deletion mutant of RS105; Rif^r^ | This study |
| RΔxrp4 | *XOC_3955* deletion mutant of RS105; Rif^r^ | This study |
| RΔxrp5 | *XOC_1550* deletion mutant of RS105; Rif^r^ | This study |
| RΔxrp6 | *XOC_3440* deletion mutant of RS105; Rif^r^ | This study |
| RΔxrp7 | *XOC_4583* deletion mutant of RS105; Rif^r^ | This study |
| RΔxrp8 | *XOC_2462* deletion mutant of RS105; Rif^r^ | This study |
| RΔxrp9 | *XOC_1951* deletion mutant of RS105; Rif^r^ | This study |
| RΔxrp10 | *XOC_0560* deletion mutant of RS105; Rif^r^ | This study |
| RΔxrp11 | *XOC_3130* deletion mutant of RS105; Rif^r^ | This study |
| RΔxrp12 | *XOC_4010* deletion mutant of RS105; Rif^r^ | This study |
| RΔxrp13 | *XOC_0860* deletion mutant of RS105; Rif^r^ | This study |
| RΔxrp14 | *XOC_0084* deletion mutant of RS105; Rif^r^ | This study |
| RΔxrp15 | *XOC_2829* deletion mutant of RS105; Rif^r^ | This study |
| RΔxrp16 | *XOC_2828* deletion mutant of RS105; Rif^r^ | This study |
| RΔxrp17 | *hrpFB* deletion mutant of strain RS105; Rif^r^ | This study |
| ***X. oryzae* pv*. oryzae*** | |  |
| PXO99^A^ | Wild-type, Philippine race 6, causes bacterial blight of rice | This lab |
| PΔ*hrcU* | *hrcU* deletion mutant of strain PXO99^A^ | This lab |
| ***E. coli*** |  |  |
| DH5α | *recA1 hsdR17*(r_K_^—^ m_K_^+^) *phoA supE44 λ^—^ thi-l gyrA96 relA1* | Invitrogen |
| S17-1 | *recA* chromosomally-integrated RP4 derivative; Sp^r^ | This lab |
| **Plasmids** |  |  |
| pMD18-T | pUC *oriV*, cloning vector, Ap^r^ | Takara |
| pUFR034 | *IncW, Mob(p)*, *Mob^+^*, *LacZa^+^*, PK2 replicon, cosmid; Km^r^ | This lab |
| pUFR034GUS | Promoterless *gusA* gene cloned in pUFR034; Km^r^ | This study |
| pxrp1GUS | pUFR034 expressing *gusA* under the *XOC_1601* promoter | This study |
| pxrp2GUS | pUFR034 expressing *gusA* under the *XOC_4584* promoter | This study |
| pxrp3GUS | pUFR034 expressing *gusA* under the *XOC_3956* promoter | This study |
| pxrp4GUS | pUFR034 expressing *gusA* under the *XOC_3955* promoter | This study |
| P*xrp5*GUS | pUFR034 expressing *gusA* under the *XOC_1550* promoter | This study |
| pxrp6GUS | pUFR034 expressing *gusA* under the *XOC_3440* promoter | This study |
| pxrp7GUS | pUFR034 expressing *gusA* under the *XOC_4583* promoter | This study |
| pxrp8GUS | pUFR034 expressing *gusA* under the *XOC_2462* promoter | This study |
| pxrp9GUS | pUFR034 expressing *gusA* under the *XOC_1951* promoter | This study |
| pxrp10GUS | pUFR034 expressing *gusA* under the *XOC_0560* promoter | This study |
| pxrp11GUS | pUFR034 expressing *gusA* under the *XOC_3130* promoter | This study |
| pxrp12GUS | pUFR034 expressing *gusA* under the *XOC_4010* promoter | This study |
| pxrp13GUS | pUFR034 expressing *gusA* under the *XOC_0860* promoter | This study |
| pxrp14GUS | pUFR034 expressing *gusA* under the *XOC_0084* promoter | This study |
| pxrp15GUS | pUFR034 expressing *gusA* under the *XOC_2829* promoter | This study |
| pxrp16GUS | pUFR034 expressing *gusA* under the *XOC_*2828 promoter | This study |
| pxrp17GUS | pUFR034 expressing *gusA* under the *HrFB* promoter | This study |
| pKMS1 | Suicide vector derived from pK18mobGII, *sacB^+^*, Km^r^ | This lab |
| pKMSΔxrp1 | Fusion of fragments flanking *XOC_*1601 in pKMS1; Km^r^ | This study |
| pKMSΔxrp2 | Fusion of fragments flanking *XOC_4584* in pKMS1; Km^r^ | This study |
| pKMSΔxrp3 | Fusion of fragments flanking *XOC_3956* in pKMS1; Km^r^ | This study |
| pKMSΔxrp4 | Fusion of fragments flanking *XOC_3955* in pKMS1; Km^r^ | This study |
| pKMSΔxrp5 | Fusion of fragments flanking *XOC_1550* in pKMS1; Km^r^ | This study |
| pKMSΔxrp6 | Fusion of fragments flanking *XOC_3440* in pKMS1; Km^r^ | This study |
| pKMSΔxrp7 | Fusion of fragments flanking *XOC_4583* in pKMS1; Km^r^ | This study |
| pKMSΔxrp8 | Fusion of fragments flanking *XOC_2462* in pKMS1; Km^r^ | This study |
| pKMSΔxrp9 | Fusion of fragments flanking *XOC_1951* in pKMS1; Km^r^ | This study |
| pKMSΔxrp10 | Fusion of fragments flanking *XOC_0560* in pKMS1; Km^r^ | This study |
| pKMSΔxrp11 | Fusion of fragments flanking *XOC_3130* in pKMS1; Km^r^ | This study |
| pKMSΔxrp12 | Fusion of fragments flanking *XOC_4010* in pKMS1; Km^r^ | This study |
| pKMSΔxrp12 | Fusion of fragments flanking *XOC_0860* in pKMS1; Km^r^ | This study |
| pKMSΔxrp14 | Fusion of fragments flanking *XOC_0084* in pKMS1; Km^r^ | This study |
| pKMSΔxrp15 | Fusion of fragments flanking *XOC_2829* in pKMS1; Km^r^ | This study |
| pKMSΔxrp16 | Fusion of fragments flanking *XOC_2828* in pKMS1; Km^r^ | This study |
| pKMSΔxrp17 | Fusion of fragments flanking *hrpFB* in pKMS1; Km^r^ | This study |
| pUFR034Myc | pUFR034 expressing c-Myc tag; Km^r^ | This study |
| pXrp1Myc | *XOC_1601* promoter and coding region cloned in-frame with c-Myc tag in pUFR034Myc, Km^r^ | This study |
| pXrp2Myc | *XOC_4584* promoter and coding region cloned in-frame with c-Myc tag in pUFR034Myc, Km^r^ | This study |
| pXrp3Myc | *XOC_*3956 promoter and coding region cloned in-frame with c-Myc tag in pUFR034Myc, Km^r^ | This study |
| pXrp4Myc | *XOC_3955* promoter and coding region cloned in-frame with c-Myc tag in pUFR034Myc, Km^r^ | This study |
| pXrp5Myc | *XOC_*1550 promoter and coding region cloned in-frame with c-Myc tag in pUFR034Myc, Km^r^ | This study |
| pXrp6Myc | *XOC_3440* promoter and coding region cloned in-frame with c-Myc tag in pUFR034Myc, Km^r^ | This study |
| pXrp7Myc | *XOC_*4583 promoter and coding region cloned in-frame with c-Myc tag in pUFR034Myc, Km^r^ | This study |
| pXrp8Myc | *XOC_2462* promoter and coding region cloned in-frame with c-Myc tag in pUFR034Myc, Km^r^ | This study |
| pXrp9Myc | *XOC_*1951 promoter and coding region cloned in-frame with c-Myc tag in pUFR034Myc, Km^r^ | This study |
| pXrp10Myc | *XOC_0560* promoter and coding region cloned in-frame with c-Myc tag in pUFR034Myc, Km^r^ | This study |
| pXrp11Myc | *XOC_*3130 promoter and coding region cloned in-frame with c-Myc tag in pUFR034Myc, Km^r^ | This study |
| pXrp12Myc | *XOC_4010* promoter and coding region cloned in-frame with c-Myc tag in pUFR034Myc, Km^r^ | This study |
| pXrp13Myc | *XOC_*0860 promoter and coding region cloned in-frame with c-Myc tag in pUFR034Myc, Km^r^ | This study |
| pXrp14Myc | *XOC_0084* promoter and coding region cloned in-frame with c-Myc tag in pUFR034Myc, Km^r^ | This study |
| pXrp15Myc | *XOC_2829* promoter and coding region cloned in-frame with c-Myc tag in pUFR034Myc, Km^r^ | This study |
| pXrp16Myc | *XOC_*2828 promoter and coding region cloned in-frame with c-Myc tag in pUFR034Myc, Km^r^ | This study |
| pXrp17Myc | *hrpFB* promoter and coding region cloned in-frame with c-Myc tag in pUFR034Myc, Km^r^ | This study |
| pBluescript II KS | Cloning vector, Ap^r^ | This lab |
| pHM1 | *IncW, Mob(p)*, *Mob^+^*, *LacIP^+^*, PK2 replicon, cosmid; Sp^r^ Sm^r^ | This lab |
| pBAvrXa10Δ28 | Contains *avrXa10* with a deletion at the 5’ end that eliminates the first 28 amino acids; cloned in pBluescript II KS with a c-Myc tag; Ap^r^ | This lab |
| pBAvrXa10 | *avrXa10* cloned in pBluescript II KS with a c-Myc tag; Ap^r^ | This study |
| pHBAvrXa10Δ28 | pBAvrXa10Δ28 cloned in pHM1; Ap^r^ Sp^r^ | This study |
| pHBAvrXa10 | pBAvrXa10 cloned in pHM1; Ap^r^ Sp^r^ | This study |
| pXrp3AvrXa10Δ28 | pHBAvrXa10Δ28 expressing the N-terminal 50 amino acids of XOC_3956 in frame with AvrXa10Δ28; expressed from the XOC_3956 promoter; Ap^r^ Sp^r^ | This study |
| pXrp5AvrXa10Δ28 | pHBAvrXa10Δ28 expressing the N-terminal 50 amino acids of XOC_1550 and AvrXa10Δ28; expressed from the XOC_1550 promoter; Ap^r^ Sp^r^ | This study |
| pA7-YFP | Derived from pUC18; encodes *yfp* under control of the CaMV 35S promoter; Ap^r^ | This lab |
| pXrp3-YFP | pA7-YFP expressing XOC_3956*;* Ap^r^ | This study |
| pXrp5-YFP | pA7-YFP expressing XOC_1550; Ap^r^ | This study |

Ap^r^ = ampicillin resistance, Km^r^ = kanamycin resistance, Rif^r^ = rifampicin resistance, Sp^r^= spectinomycin resistance

**Table S2.** Oligonucleotide primers used in this study

| **Primers** | **Sequence (5’→3’; restriction sites underlined)** | | **Description** |
| --- | --- | --- | --- |
| **q-RT** |  | |  |
| *gyrb*-q-F | CGGCACTTACGACTCCAGCAA | | A 191 bp fragment of *XOC_0006* |
| *gyrb* -q-R | CGACCAGGATTTTCACCACGA | |
| *rpoD*-q-F | CGACAACACCACCAACATCAATC | | A 140 bp fragment of *XOC_2329* |
| *rpoD*-q-R | GCTTACCGACCTCTTCCAACG | |
| xrp1-q-F | GACACCGAGCAGCATTAC | | A 209 bp fragment of *XOC_1601* |
| xrp1-q-R | CGCACATAGAAGCCGATC | |
| xrp2-q-F | TGCATACCGTCGCCAAAGC | | A 195 bp fragment of *XOC_4584* |
| xrp2-q-R | GACATCGTCGTCAGTGTTCAGG | |
| xrp3-q-F | CCTGGAGTATCGCTATTCG | | A 199 bp fragment of *XOC_3956* |
| xrp3-q-R | TCATGCCTGGACATAACG | |
| xrp4-q-F | GACTCCGCACGCTCTACC | | A 160 bp fragment of *XOC_3955* |
| xrp4-q-R | TCCAGACGCACCACAAGG | |
| xrp5-q-F | GTGCCGATTACCTGGTCTG | | A 173 bp fragment of *XOC_1550* |
| xrp5-q-R | CTTGTTGCCGCCGAGATC | |
| xrp6-q-F | TCGCAGACGTTGTCCGAAAGC | | A 106 bp fragment of *XOC_3440* |
| xrp6-q-R | GCATACCGCCAGCAGTGTCG | |
| xrp7-q-F | ATGCGTTCTTCCGTGTTGTC | | A 183 bp fragment of *XOC_4583* |
| xrp7-q-R | CAGCAATTCGGTAGCGTCAG | |
| xrp8-q-F | AAGGACGCAATGGGCAAGCT | | A 113 bp fragment of *XOC_2462* |
| xrp8-q-F | ATCTCCAGGGTCTCGAACAC | |
| xrp9-q-R | ACACGGCGGCTTCTATTTCG | | A 231 bp fragment of *XOC_1951* |
| xrp9-q-R | ATCTCGCGGTGGTCTTGCA | |
| xrp10-q-F | ACCGAAGAGCCGTATTACACCGAA | | A 133 bp fragment of *XOC_0560* |
| xrp10-q-R | AATCGCGATGCAACTCGGCTTCAA | |
| xrp11-q-F | CAAAACCGAACATAGCCTCA | | A 236 bp fragment of *XOC_3130* |
| xrp11-q-R | GCACCTTTCCGTAGAACTGC | |
| xrp12-q-F | CGTGGTGATTCCATTCGTG | | A 119 bp fragment of *XOC_4010* |
| xrp12-q-R | GCATGTCCTTGTGATTGAGC | |
| xrp13-q-F | AACAGATCCTGCAAGTGGGATTGG | | A 173 bp fragment of *XOC_0860* |
| xrp13-q-R | CAGATATACCTTGACCTGCGCGAT | |
| xrp14-q-F | CACATCCCAGCCGAAAGCAA | | A 196 bp fragment of *XOC_0084* |
| xrp14-q-R | GGAAAATCGAACCGAACACCAC | |
| xrp15-q-F | GCACCGCCAGCTCAATCAAT | | A 177 bp fragment of *XOC_2829* |
| xrp15-q-R | AGCACCATCAGCACGCAATC | |
| xrp16-q-F | GCAGATCGGTGCCAACTGTTTCAT | | A 168 bp fragment of *XOC_2828* |
| xrp16-q-R | ATCTCGCAATAGCCGGAAATCACC | |
| xrp17-q-F | TGGAGATGCAGATCATCGTGGTGT | | A 111 bp fragment of *hrpFB* |
| xrp17-q-R | ATCCAGTCAGAACAGCCCTCACAA | |
| **Mutagenesis** |  | |  |
| xrp1I-F | ATAGGATCCCCCTACCTCGCCCTGGTCGCC | | The left homologous fragment for  *XOC_1601* knock-out, 230 bp |
| xrp1I-R | TATCCCGGGGTTTCCTTCGCATGAACACGC | |
| xrp1II-F | ATAGTCGACGGCCTTGGTCTGCCGTTCGTG | | The right homologous fragment for *XOC_1601* knock-out, 350 bp |
| xrp1II-R | TATGGATCCATCGTGGAAAACAGCTGGGGC | |
| xrp2 I-F | CGGGATCCTGTTTCCGTAATCACAATCGGCCTT | | The left homologous fragment for  *XOC_4584* knock-out, 686 bp |
| xrp2I-R | AAAAACGCCCGGGAAGGCCTGGTACCCCTAAGCATCCTCATTGGGAGAGCGAAAG | |
| xrp2II-F | GGGGTACCAGGCCTTCCCGGGCGTTTTTTGTTG | | The right homologous fragment for *XOC_4584* knock-out, 342 bp |
| xrp2II-R | GGTTCTAGAGGGGTTTTTCAGGGGCGACT | |
| xrp3I-F | TCCCCCGGGGCCAGGCGGTAGAGC | | The left homologous fragment for  *XOC_3956* knock-out, 343 bp |
| xrp3I-R | GGGGTACCAGTATCGCTATTCGC | |
| xrp3II-F | GGGGTACCAGGGTTATGGCCGTGC | | The right homologous fragment for *XOC_3956* knock-out, 248 bp |
| xrp3II-R | CGGGATCCTCCGAGCACCTGCAACGC | |
| xrp4 I-F | TGCTCTAGAGGACAGACCGGTGTAGCGCTCGAAC | | The left homologous fragment for  *XOC_3955* knock-out, 500 bp |
| xrp4I-R | GCGCCTAGGGCCTGTTGCTGCTGGATTCGGACTC | |
| xrp4II-F | CGCGGATCCGTTCATGCCTGGACATAACGGGGAC | | The right homologous fragment for *XOC_3955* knock-out, 500 bp |
| xrp4II-R | AGGGGGCCCTGGCCGTGCAGTTCCCGCCGGTCTA | |
| xrp5I-F | CGCGGATCCCCAAGCAGTGGATAGCGCC | | The left homologous fragment for  *XOC_1550* knock-out, 451 bp |
| xrp5I-R | CTAGTCTAGACGCAGCCTGCGTGACAGC | |
| xrp5II-F | CTAGTCTAGAACACGATTCCCTGGCATT | | The right homologous fragment for *XOC_1550* knock-out, 695 bp |
| xrp5II-R | ACGCGTCGACGCGTGTGGCTGCGCGTGA | |
| xrp*6*I-F | ACGCGTCGACATGCCGGTCTCTCTCTTGGGGG | | The left homologous fragment for  *XOC_3440* knock-out, 250 bp |
| xrp6I-R | GCTCTAGAAGACCAATGGCATCGATATCCG | |
| xrp6II-F | GCTCTAGACCGACGCCCACATGCCCGCTAT | | The right homologous fragment for *XOC_3440* knock-out, 350 bp |
| xrp6II-R | CGCGGATCCGCGAGACCAGGCGGGCGTCCTG | |
| xrp7I-F | ATACCCGGGCAGCGCTTCTTCCCGATGTTC | | The left homologous fragment for  *XOC_4583* knock-out, 403 bp |
| xrp7I-R | TATGGATCCGCACCAACAGCAATTCGGTAG | |
| xrp7II-F | ATAGGATCCTCGCCAAACTGGTTGCCGAGC | | The right homologous fragment for *XOC_4583* knock-out, 252 bp |
| xrp7II-R | TATGTCGACCGCGGCGACGAAGGCATAGCT | |
| xrp8 I-F | AATGGATCCCATCGCGAGACGTTTATC | | The left homologous fragment for  *XOC_2462* knock-out, 607 bp |
| xrp8I-R | ATATCTAGACCGTCGCATGCGCGTC | |
| xrp8II-F | AATTCTAGACGGTGTTACCGCGAAGAAGT | | The right homologous fragment for *XOC_2462* knock-out, 310 bp |
| xrp8II-R | TATGTCGACTGCACATGGGCAATGGTTCC | |
| xrp9I-F | TATGGATCCGGACAATCAGTATCTGGTTG | | The left homologous fragment for  *XOC_1951* knock-out, 746 bp |
| xrp9I-R | ATATCTAGATCCGTTCGTAGCGCTCG | |
| xrp9II-F | TATTCTAGAAGTTGAACGCTGTGCCGATT | | The right homologous fragment for *XOC_1951* knock-out, 330 bp |
| xrp9II-R | ATTGTCGACTGTGTGCAGCCTGGG | |
| xrp10 I-F | TGCTCTAGAGGTTGTGGAATGCCCAGAT | | The left homologous fragment for  *XOC_0560* knock-out, 407 bp |
| xrp10I-R | AACTGCAGAACGACAACGCCACCGT | |
| xrp10II-F | AACTGCAGGCGCACGTCTCCGTG | | The right homologous fragment for *XOC_0560* knock-out, 732 bp |
| xrp10II-R | ACATGCATGCGCAGGCTTGGCGCT | |
| xrp11I-F | ATAGGATCCTACAGGAACTGTTGCTTCATG | | The left homologous fragment for  *XOC_3130* knock-out, 330 bp |
| xrp11I-R | TATTCTAGAGCGTAACCGCAAGCATGCAAT | |
| xrp11II-F | ATATCTAGACTATGTTCGGTTTTGCGCTGA | | The right homologous fragment for *XOC_3130* knock-out, 839 bp |
| xrp11II-R | TATCTGCAGCCTTGCAGACACGCTTGATGG | |
| xrp12 I-F | CGCGGATCCCATTTTGCTGCCGGCCTGTGCAT | | The left homologous fragment for  *XOC_4010* knock-out, 467 bp |
| xrp12I-R | GCTCTAGACAGGATCAGGTCGTCGCGTTCG | |
| xrp*12*II-F | GCTCTAGACGGCGATGCGTTCGACAAGGGC | | The right homologous fragment for *XOC_4010* knock-out, 242 bp |
| xrp12II-R | GCGTCGACCGCGTCGCCTGCGTTTGCAACC | |
| xrp13I-F | TCCCCCGGGGCCTGGCGGCGGAAGTGGAGCGGGT | | The left homologous fragment for  *XOC_0860* knock-out, 443 bp |
| xrp13I-R | GCGGGATCCCGCGATGGCGGAGGCAAGTACGGCC | |
| xrp13II-F | CGCGGATCCGTGTCAATGCGCATGGCGCGCTCGC | | The right homologous fragment for *XOC_0860* knock-out, 728 bp |
| xrp13II-R | CGAGATCTCGAGACATCGCTCCTGCTTGTTTTG | |
| xrp14I-F | CGGGATCCCGGTTCCGACGCCAGT | | The left homologous fragment for  *XOC_0084* knock-out, 675 bp |
| xrp14I-R | GGGGTACCTCATCACCGGCGGCTT | |
| xrp14II-F | GGGGTACCCAGCATGGCAGGC | | The right homologous fragment for *XOC_0084* knock-out, 494 bp |
| xrp14II-R | TCCCCCGGGTGGTCGCAGGCAATC | |
| xrp15I-F | TTTGGATCCAGCACCGATGATGTTGTTTG | | The left homologous fragment for  *XOC_2829* knock-out, 658 bp |
| xrp15I-R | TTGCATATGACCCGGGCGATTACATCCTGGA | |
| xrp15II-F | CCCATATGGGTGTCCGCGCAAGCGCAATCGAAC | | The right homologous fragment for *XOC_2829* knock-out, 499 bp |
| xrp15II-R | GCTCTAGAGCGCAGACGCAGGCGTAACCCGATG | |
| xrp16I-F | GTCGGATCCGGTGGTGGTCATTGGTT | | The left homologous fragment for  *XOC_2828* knock-out, 737 bp |
| xrp16I-R | GATTCTAGAATTGTTTGGGCGCGCTCATGC | |
| xrp16II-F | CAGTCTAGACGTGGAGCTCTGACCCGTGTTT | | The right homologous fragment for *XOC_2828* knock-out, 847 bp |
| xrp16II-R | TTTCTGCAGGGCCGGGTAGCACAGTGTCT | |
| xrp17I-F | AAACTGCAGCGGCCAGGGTGCACG | | The left homologous fragment for  *hrpFB* knock-out, 751 bp |
| xrp17I-R | ACATGCATGCAAATCCCACAGATC | |
| xrp17II-F | TGCTCTAGAGATTCCAAGATCATC | | The right homologous fragment for *hrpFB* knock-out, 451 bp |
| xrp17II-R | AAACTGCAGCATTTCTTCTATTTC | |
| **Western and Complemented**  **Construction** | |  |  |
| xrp1-F | CGAGCTCTTTCATCGGCCATCCTCATCGTG | | A 1226 bp fragment containing the entire *XOC_1601* gene and its own promoter |
| xrp1-R | CGAGCTCGTGGACCACGTCAGCGACGATG | |
| xrp2-F | CGAGCTCTGCTCGGCCCCGCATGGTGGTTC | | A 1556 bp fragment containing the entire *XOC_4584* gene and its own promoter |
| xrp2-R | CGGGGTACCGTTGCTTTCAGTAAAGGTGATG | |
| xrp3-F | CGAGCTCGCGTATAGGTGAAACTGATCCG | | A 1092 bp fragment containing the entire *XOC_3956* gene and its own promoter |
| xrp3-R | CGAGCTCTGCCTGGACATAACGGGGACGC | |
| xrp4-F | CGAGCTCCCCGGAAGATCGCCAGCATGGAC | | A 806 bp fragment containing the entire *XOC_3955* gene and its own promoter |
| xrp4-R | CGGGGTACCTTGCCAATTCCAGACGCACCAC | |
| xrp5-F | CGGGGTACCCACCTGATCGTGCCCTCGCCCG | | A 1001 bp fragment containing the entire *XOC_1550* gene and its own promoter |
| xrp5-R | CGGGGTACCGCGCTGCAGCGCCAGACTGGC | |
| xrp6-F | CGGGGTACCTGTCATTGGCGCAGTCGAAAC | | A 913 bp fragment containing the entire *XOC_3440* gene and its own promoter |
| xrp6-R | CGGGGTACCAGCCAGCACGTCGTGAATTTCGC | |
| xrp7-F | CGGGGTACCTGCTTGAGGCATTCCGCCTGGC | | A 1923 bp fragment containing the entire *XOC_4583* gene and its own promoter |
| xrp7-R | CGGGGTACCCAGGTTGTAGGCCCGCTTGGGCA | |
| xrp8-F | CGAGCTCGCCTGGGTAGCTTTGAAGGTCCAC | | A 983 bp fragment containing the entire *XOC_2462* gene and its own promoter |
| xrp8-F | CGGGGTACCCTTCTTCGCGGTAACACCGGAGG | |
| xrp9-R | CGAGCTCCTGCCTGGCAATGCTGTGCTGCCA | | A 1067 bp fragment containing the entire *XOC_1951* gene and its own promoter |
| xrp9-R | CGGGGTACCAATCGGCACAGCGTTCAACTTGC | |
| xrp10-F | CGAGCTCCCACGCCCGCCTGGCGGCTGCTCG | | A 830 bp fragment containing the entire *XOC_0560* gene and its own promoter |
| xrp10-R | CGGGGTACCGCGCTTATGTGTATCGTCGCCGA | |
| xrp11-F | CGAGCTCAATTCGCATCGCTGTGCGGTTGT | | A 953 bp fragment containing the entire *XOC_3130* gene and its own promoter |
| xrp11-R | CGGGGTACCTGCGGCGCTCTTCATTGCATGCT | |
| xrp12-F | CGGGGTACCAGTGTGCGCTCGTTCTGATTGAAG | | A 1661 bp fragment containing the entire *XOC_4010* gene and its own promoter |
| xrp12-R | CGGGGTACCCGGCGCGAGCACGACCTTGCGA | |
| xrp13-F | CGGGGTACCAAGCAGGCGGTCATCACCGGCTC | | A 2207 bp fragment containing the entire *XOC_0860* gene and its own promoter |
| xrp13-R | CGGGGTACCTGGCGATGCATGCGTCAGGAAG | |
| xrp14-F | CGAGCTCCGGCGTCATTGGCAATGGTGTGC | | A 1700 bp fragment containing the entire *XOC_0084* gene and its own promoter |
| xrp14-R | CGGGGTACCCCAGCGAACCGGGAACGCCTCA | |
| xrp15-F | CGAGCTCGCACGGCCATCCAGTTCGGCGCG | | A 929 bp fragment containing the entire *XOC_2829* gene and its own promoter |
| xrp15-R | CGGGGTACCTGCTTCCAGCCTGCAAGCGTGC | |
| xrp16-F | CGGGGTACCTGCGTCGCCTCTGCACATCGCTC | | A 1271 bp fragment containing the entire *XOC_2828* gene and its own promoter |
| xrp16-R | CGGGGTACCGAGCTCCACGTCGAAGCTGGA | |
| xrp17-F | CGAGCTCTCGACCCCTCCAGCGCCGGCGGC | | A 740 bp fragment containing the entire *hrpFB* gene and its own promoter |
| xrp17-R | CGGGGTACCTGCAACCAGGTTCCATGAAAGC | |
| **Reporter construction** |  | |  |
| *gusA*-F | TTTGAGCTCATGTTACGTCCTGTAGAAAC | | A 1812 bp fragment containing the entire *gusA* gene |
| *gusA-*R | CGGGGTACCTCATTGTTTGCCTCCCTGCTGC | |
| pxrp1-F | CGGGGTACCTTTCATCGGCCATCCTCATCG | | A 500 bp fragment of *XOC_1601* promoter |
| pxrp1-R | TTTGAGCTCTCCGTGTCCTGCCGCCTGTGC | |
| pxrp2-F | CGGGGTACCTGCTCGGCCCCGCATGGTGGT | | A 500 bp fragment of *XOC_4584* promoter |
| pxrp2-R | TTTGAGCTCTTGCCCGCCTCATGTAGATGG | |
| pxrp3-F | CGCGGATCCGCGTATAGGTGAAACTGATCC | | A 500 bp fragment of *XOC_3956* promoter |
| pxrp3-R | TTTGAGCTCGCTGGCGATCTTCCGGGCCGT | |
| pxrp4-F | CGGGGTACCCCCGGAAGATCGCCAGCATGG | | A 500 bp fragment of *XOC_3955* promoter |
| pxrp4-R | TTTGAGCTCAACGGGGACGCTCCTGATCAA | |
| pxrp5-F | CGCGGATCCCACCTGATCGTGCCCTCGCCC | | A 500 bp fragment of *XOC_1550* promoter |
| pxrp5-R | CGGGGTACCACACGATTCCCTGGCATTGGA | |
| pxrp6-F | CGCGGATCCGGATGTCATTGGCGCAGTCGA | | A 500 bp fragment of *XOC_3440* promoter |
| pxrp6-R | CGGGGTACCTGACCGTTATTGGCCGCAGCC | |
| pxrp7-F | CGGGGTACCAGGCATTCCGCCTGGCACGCA | | A 500 bp fragment of *XOC_4583* promoter |
| pxrp7-R | TTTGAGCTCGATCGATATCAGCCCAATGGA | |
| pxrp8-F | CGGGGTACCCGATTGCACATTTTTAAAAGT | | A 500 bp fragment of *XOC_2462* promoter |
| pxrp8-F | TTTGAGCTCCAGGTTTTCTTGGGGTTCGGCCT | |
| pxrp9-R | CGGGGTACCGCGGATCGGTGTTGGAAATGAGC | | A 500 bp fragment of *XOC_1951* promoter |
| pxrp9-R | TTCGAGCTCCAGTTCTGAGTTATCGGCGTCGT | |
| pxrp10-F | CGGGGTACCAGTGGACGCGTGGTCCATGCCGC | | A 500 bp fragment of *XOC_0560* promoter |
| pxrp10-R | TTCGAGCTCGCGCACGTCTCCGTGGTGGATGT | |
| pxrp11-F | CGCGGATCCGCCAGCAGCGCTCGGTCTTGGC | | A 500 bp fragment of *XOC_3130* promoter |
| pxrp11-R | GCTCTAGAGTGCGGCTCCTTTGCTGGAATG | |
| pxrp12-F | CGGGGTACCCTCCTTTCACCGCCTTCACCCC | | A 500 bp fragment of *XOC_4010* promoter |
| pxrp12-R | TTTGAGCTCGTGTCTCTCCTGTAGGCGTGAT | |
| pxrp13-F | CGGGGTACCGCCACCATCGAACTGCCGATTCC | | A 500 bp fragment of *XOC_0860* promoter |
| pxrp13-R | TTCGAGCTCCGCGATGGCGGAGGCAAGTACGG | |
| pxrp14-F | CGGGGTACCATCGCCGGGCGCGACAACGGCC | | A 500 bp fragment of *XOC_0084* promoter |
| pxrp14-R | TTCGAGCTCGCGCTCACTCTCTAGACGGCAG | |
| pxrp15-F | CGCGGATCCGCGTGCGCAGACGCAGGCGTAAC | | A 500 bp fragment of *XOC_2829* promoter |
| pxrp15-R | GCTCTAGAGATCGGAGAGGCGTACCGCTAAC | |
| pxrp16-F | CGGGGTACCTCGCCTCTGCACATCGCTCGCAC | | A 500 bp fragment of *XOC_2828* promoter |
| pxrp16-R | TTCGAGCTCGCTTCCAGCCTGCAAGCGTGCGG | |
| pxrp17-F | CGGGGTACCACTTGGTCAGCGTGCCATCGTTC | | A 500 bp fragment of *hrpFB* promoter |
| pxrp17-R | TTCGAGCTCCGGCCAGGGTGCACGTTGCGCAAAT | |
| xrp3-N-F | CGGGAGCTCGCGTATAGGTGAAACTGATCC | | The N-terminal of *XOC_3956* gene (711 bp) |
| xrp3-N-R | AAAACTGCAGTCTCGCTGTGCAGGGCGACGA | |
| xrp5-N-F | AAAACTGCAGACCTGATCGTGCCCTCGCCCGA | | The N-terminal of *XOC_1550* gene (651 bp) |
| xrp5-N-R | AAAACTGCAGTCAGGCCGGAGCCCGGCGGCA | |
| **Subcellular loacalization** |  | |  |
| xrp3-Y-F | CGCGTCGACATGGACACTCGCTCCTTCCACA | | A 495 bp fragment containing the entire *XOC_3956* gene |
| xrp3-Y-R | CGCGGATCCTGCCTGGACATAACGGGGACGC | |
| xrp5-Y-F | CGCGTCGACATGGGCGGGGAGCTCAAGGCACTG | | A 579 bp fragment containing the entire *XOC_1550* gene |
| xrp5-Y-R | CGCGGATCCGCGCTGCAGCGCCAGACTGGC | |

**Table S3.** Differentially expression profiles of hypothetical protein genes in the *hrpG* and *hrpX* mutants R*∆hrpG* and R*∆hrpX* compared to the wild-type RS105 tested by microarray assay

| ***Xoc* Locus ID^a^** | **ratio (R∆*hrpG***  **/RS105)^b^** | | **P-value** | **ratio (R∆*hrpX*/RS105)** | | **P-value** | **Gene annotation** |
| --- | --- | --- | --- | --- | --- | --- | --- |
| **Hypothetical protein** | | | | | | | |
| ZP_02242251 | 0.567 | | 5.941E-06 | 12.382 | 3.353E-06 | | hypothetical protein |
| ZP_02241802 | 0.507 | | 2.831E-06 | 9.846 | 7.327E-06 | | hypothetical protein |
| ZP_02242629 | 3.292 | | 1.656E-06 | 8.522 | 1.464E-05 | | hypothetical protein |
| ZP_02242031 | 0.485 | | 7.964E-06 | 8.413 | 1.071E-07 | | hypothetical protein |
| ZP_02241484 | 2.390 | | 2.938E-06 | 7.813 | 8.207E-06 | | hypothetical protein |
| ZP_02243800 | 1.332 | | 2.638E-05 | 7.768 | 1.160E-05 | | hypothetical protein |
| ZP_02245362 | 1.078 | | 8.174E-03 | 7.616 | 1.478E-05 | | hypothetical protein, Xrp7^c^ |
| ZP_02244344 | 1.418 | | 8.914E-05 | 6.472 | 3.649E-06 | | hypothetical protein |
| ZP_02243647 | 1.382 | | 4.497E-06 | 4.813 | 8.124E-06 | | hypothetical protein |
| ZP_02241142 | 1.078 | | 5.995E-06 | 4.202 | 5.662E-06 | | hypothetical protein |
| ZP_02241135 | 1.078 | | 1.265E-05 | 3.981 | 2.809E-06 | | hypothetical protein |
| ZP_02243583 | 1.803 | | 9.037E-06 | 3.746 | 3.716E-06 | | hypothetical protein |
| ZP_02244140 | 0.804 | | 3.192E-06 | 3.337 | 4.192E-05 | | hypothetical protein |
| ZP_02241413 | 0.714 | | 2.881E-05 | 3.226 | 1.458E-05 | | hypothetical protein |
| ZP_02242519 | 1.078 | | 1.408E-04 | 3.156 | 4.760E-06 | | hypothetical protein |
| ZP_02242260 | 1.078 | | 5.003E-05 | 3.118 | 9.875E-06 | | hypothetical protein |
| ZP_02242262 | 1.450 | | 3.328E-05 | 3.095 | 1.861E-05 | | hypothetical protein |
| ZP_02245393 | 1.332 | | 6.666E-06 | 2.948 | 5.316E-06 | | hypothetical protein |
| ZP_02244093 | 1.078 | | 2.815E-06 | 2.931 | 5.095E-06 | | hypothetical protein |
| ZP_02243185 | 4.893 | | 1.926E-05 | 2.861 | 5.212E-06 | | hypothetical protein |
| ZP_02243715 | 0.729 | | 7.703E-06 | 2.798 | 2.077E-05 | | hypothetical protein, Xrp15 |
| ZP_02242847 | 2.636 | | 6.341E-06 | 2.772 | 1.152E-05 | | hypothetical protein |
| ZP_02244922 | 0.546 | | 9.462E-06 | 2.759 | 6.929E-06 | | hypothetical protein |
| ZP_02241399 | 1.246 | | 2.452E-05 | 2.726 | 3.649E-06 | | hypothetical protein |
| ZP_02243590 | 1.249 | | 7.074E-06 | 2.699 | 9.018E-06 | | hypothetical protein |
| ZP_02243719 | 1.790 | | 7.742E-06 | 2.613 | 9.412E-06 | | hypothetical protein |
| ZP_02242225 | 0.527 | | 9.057E-06 | 2.586 | 1.130E-05 | | hypothetical protein |
| ZP_02244846 | 0.999 | | 2.527E-06 | 2.571 | 1.111E-05 | | hypothetical protein |
| ZP_02245216 | 2.267 | | 2.494E-06 | 2.539 | 9.511E-06 | | hypothetical protein |
| ZP_02242001 | 0.898 | | 3.494E-06 | 2.537 | 1.151E-05 | | hypothetical protein |
| ZP_02242925 | 0.697 | | 2.987E-05 | 2.500 | 9.957E-06 | | hypothetical protein |
| ZP_02245248 | 0.902 | | 8.081E-03 | 2.452 | 4.588E-05 | | hypothetical protein, Xrp17 |
| ZP_02242592 | 1.710 | | 2.335E-05 | 2.449 | 8.067E-07 | | hypothetical protein |
| ZP_02244592 | 0.497 | | 3.125E-05 | 2.446 | 4.797E-06 | | hypothetical protein |
| ZP_02241243 | 0.796 | | 4.094E-06 | 2.443 | 3.465E-06 | | hypothetical protein |
| ZP_02241558 | 1.294 | | 7.806E-05 | 2.439 | 1.224E-06 | | hypothetical protein |
| ZP_02243445 | 1.647 | | 7.061E-05 | 2.434 | 2.646E-06 | | hypothetical protein |
| ZP_02241340 | 0.858 | | 1.146E-05 | 2.416 | 4.648E-06 | | hypothetical protein |
| ZP_02242302 | 0.238 | | 4.485E-05 | 2.386 | 6.199E-06 | | hypothetical protein |
| ZP_02244002 | 0.727 | | 2.016E-05 | 2.381 | 3.649E-06 | | hypothetical protein |
| ZP_02243276 | 3.391 | | 1.044E-06 | 2.368 | 3.812E-06 | | hypothetical protein |
| ZP_02242860 | 1.322 | | 5.734E-06 | 2.364 | 1.665E-06 | | hypothetical protein |
| ZP_02242301 | 2.220 | | 3.178E-06 | 2.292 | 4.618E-06 | | hypothetical protein |
| ZP_02243674 | 2.030 | | 5.052E-06 | 2.275 | 1.024E-06 | | hypothetical protein |
| ZP_02244779 | 1.537 | | 6.192E-06 | 2.247 | 6.897E-06 | | hypothetical protein |
| ZP_02243258 | 3.937 | | 2.729E-06 | 2.238 | 1.854E-05 | | hypothetical protein |
| ZP_02241679 | 2.001 | | 7.606E-06 | 2.236 | 5.517E-06 | | hypothetical protein |
| ZP_02242556 | 4.061 | | 4.438E-06 | 2.211 | 9.875E-06 | | hypothetical protein |
| ZP_02243257 | 3.250 | | 6.389E-06 | 2.183 | 3.353E-06 | | hypothetical protein |
| ZP_02242006 | 2.560 | | 1.353E-05 | 2.161 | 7.327E-06 | | hypothetical protein |
| ZP_02242365 | 0.704 | | 2.459E-06 | 2.160 | 1.464E-05 | | hypothetical protein |
| ZP_02242852 | 2.257 | | 8.228E-06 | 2.154 | 1.284E-05 | | hypothetical protein |
| ZP_02241402 | 0.149 | | 4.670E-06 | 2.143 | 6.234E-06 | | hypothetical protein |
| ZP_02244561 | 0.764 | | 3.275E-05 | 2.141 | 1.071E-07 | | hypothetical protein |
| ZP_02244075 | 2.788 | | 1.616E-05 | 2.134 | 8.207E-06 | | hypothetical protein |
| ZP_02241695 | 0.475 | | 1.476E-05 | 2.113 | 1.160E-05 | | hypothetical protein |
| ZP_02242643 | 0.398 | | 1.854E-05 | 2.106 | 8.779E-06 | | hypothetical protein |
| ZP_02243928 | 3.311 | | 7.603E-06 | 2.078 | 3.649E-06 | | hypothetical protein |
| ZP_02244144 | 2.199 | | 3.667E-06 | 2.071 | 1.235E-05 | | hypothetical protein |
| ZP_02244056 | 1.500 | | 2.667E-06 | 2.065 | 3.190E-06 | | hypothetical protein |
| ZP_02242311 | 2.109 | | 8.901E-06 | 2.051 | 2.836E-05 | | hypothetical protein |
| ZP_02244668 | 1.935 | | 3.374E-06 | 2.048 | 7.709E-06 | | hypothetical protein |
| ZP_02241160 | 1.203 | | 1.697E-05 | 2.035 | 1.123E-05 | | hypothetical protein |
| ZP_02244484 | 1.232 | | 6.591E-06 | 2.027 | 1.470E-06 | | hypothetical protein |
| ZP_02243079 | 1.896 | | 8.792E-06 | 2.018 | 2.442E-06 | | hypothetical protein |
| ZP_02241239 | 1.476 | | 5.238E-06 | 2.015 | 8.124E-06 | | hypothetical protein |
| ZP_02242208 | 0.522 | | 5.449E-07 | 2.015 | 5.662E-06 | | hypothetical protein |
| ZP_02241161 | 2.245 | | 1.561E-05 | 2.011 | 2.809E-06 | | hypothetical protein |
| ZP_02243765 | 2.704 | | 1.651E-06 | 2.006 | 3.716E-06 | | hypothetical protein |
| ZP_02242293 | 2.826 | | 2.181E-06 | 2.005 | 4.192E-05 | | hypothetical protein |
| ZP_02242979 | 2.335 | | 6.710E-06 | 2.002 | 1.458E-05 | | hypothetical protein |
| ZP_02244649 | 1.522 | | 6.995E-06 | 1.997 | 4.760E-06 | | hypothetical protein |
| ZP_02241216 | 3.161 | | 2.085E-06 | 1.980 | 9.875E-06 | | hypothetical protein |
| ZP_02244755 | 0.883 | | 1.755E-06 | 1.976 | 1.861E-05 | | hypothetical protein |
| ZP_02242305 | 0.681 | | 7.507E-06 | 1.976 | 5.316E-06 | | hypothetical protein |
| ZP_02243833 | 0.884 | | 2.460E-06 | 1.975 | 5.095E-06 | | hypothetical protein |
| ZP_02244914 | 1.824 | | 6.141E-06 | 1.966 | 1.084E-05 | | hypothetical protein |
| ZP_02242617 | 2.568 | | 7.421E-06 | 1.959 | 5.205E-06 | | hypothetical protein |
| ZP_02242227 | 0.872 | | 6.519E-06 | 1.953 | 5.212E-06 | | hypothetical protein |
| ZP_02242564 | 2.039 | | 3.112E-06 | 1.949 | 8.774E-06 | | hypothetical protein |
| ZP_02243565 | 4.620 | | 6.386E-06 | 1.947 | 1.152E-05 | | hypothetical protein |
| ZP_02242494 | 0.739 | | 1.448E-06 | 1.947 | 6.929E-06 | | hypothetical protein |
| ZP_02243787 | 4.720 | | 1.859E-06 | 1.947 | 3.649E-06 | | hypothetical protein |
| ZP_02243423 | 0.608 | | 9.144E-09 | 1.945 | 2.374E-06 | | hypothetical protein |
| ZP_02242646 | 0.496 | | 5.236E-06 | 1.945 | 3.195E-06 | | hypothetical protein |
| ZP_02243001 | 0.580 | | 1.333E-05 | 1.927 | 2.652E-05 | | hypothetical protein |
| ZP_02243202 | 0.625 | | 2.976E-06 | 1.924 | 2.163E-06 | | hypothetical protein |
| ZP_02241808 | 0.988 | | 4.093E-06 | 1.914 | 3.249E-06 | | hypothetical protein |
| ZP_02243424 | 1.315 | | 4.084E-06 | 1.904 | 3.483E-06 | | hypothetical protein |
| ZP_02242361 | 0.623 | | 5.363E-06 | 1.878 | 2.442E-06 | | hypothetical protein |
| ZP_02242394 | 0.986 | | 7.897E-07 | 1.872 | 9.018E-06 | | hypothetical protein |
| ZP_02243438 | 2.902 | | 3.868E-06 | 1.872 | 9.412E-06 | | hypothetical protein |
| ZP_02242914 | 3.543 | | 7.400E-06 | 1.868 | 1.130E-05 | | hypothetical protein |
| ZP_02245407 | 0.490 | | 3.316E-06 | 1.868 | 1.111E-05 | | hypothetical protein |
| ZP_02244378 | 3.265 | | 1.142E-05 | 1.866 | 9.511E-06 | | hypothetical protein |
| ZP_02245336 | 0.322 | | 3.675E-08 | 1.864 | 1.151E-05 | | hypothetical protein |
| ZP_02243139 | 1.042 | | 1.786E-06 | 1.864 | 9.957E-06 | | hypothetical protein |
| ZP_02243119 | 0.918 | | 1.971E-05 | 1.859 | 9.875E-06 | | hypothetical protein |
| ZP_02243777 | 4.189 | | 1.048E-05 | 1.844 | 8.067E-07 | | hypothetical protein |
| ZP_02245051 | 0.594 | | 3.738E-06 | 1.841 | 4.797E-06 | | hypothetical protein |
| ZP_02241258 | 1.271 | | 7.980E-06 | 1.830 | 3.465E-06 | | hypothetical protein |
| ZP_02241433 | 0.796 | | 1.344E-06 | 1.829 | 5.472E-06 | | hypothetical protein |
| ZP_02241795 | 4.348 | | 1.105E-05 | 1.823 | 9.121E-07 | | hypothetical protein |
| ZP_02243280 | 3.029 | | 2.756E-07 | 1.823 | 1.224E-06 | | hypothetical protein |
| ZP_02243130 | 0.814 | | 1.518E-05 | 1.815 | 2.646E-06 | | hypothetical protein |
| ZP_02242351 | 0.780 | | 1.323E-05 | 1.811 | 4.648E-07 | | hypothetical protein |
| ZP_02243443 | 1.532 | | 1.060E-05 | 1.807 | 6.199E-06 | | hypothetical protein |
| ZP_02243879 | 0.911 | | 1.336E-05 | 1.799 | 3.649E-06 | | hypothetical protein |
| ZP_02244849 | 4.557 | | 4.040E-06 | 1.797 | 3.125E-06 | | hypothetical protein |
| ZP_02243794 | 1.763 | | 4.643E-06 | 1.781 | 1.388E-06 | | hypothetical protein |
| ZP_02241938 | 1.142 | | 4.995E-05 | 1.779 | 5.914E-06 | | hypothetical protein |
| ZP_02244442 | 0.672 | | 4.747E-06 | 1.774 | 2.639E-06 | | hypothetical protein |
| ZP_02243926 | 3.073 | | 4.157E-06 | 1.771 | 5.860E-06 | | hypothetical protein |
| ZP_02243970 | 1.545 | | 4.214E-06 | 1.761 | 3.936E-06 | | hypothetical protein |
| ZP_02244415 | 1.552 | | 1.110E-05 | 1.758 | 2.442E-06 | | hypothetical protein |
| ZP_02242715 | 0.997 | | 5.224E-06 | 1.753 | 3.812E-04 | | hypothetical protein |
| ZP_02243714 | 1.568 | | 9.179E-06 | 0.939 | 8.057E-03 | | hypothetical protein, Xrp16 |
| ZP_02242520 | 1.558 | | 1.218E-05 | 0.899 | 5.294E-02 | | hypothetical protein, Xrp5 |
| ZP_02244768 | 0.458 | | 5.436E-06 | 0.823 | 7.682E-06 | | hypothetical protein, Xrp3 |
| ZP_02244767 | 0.549 | | 5.236E-06 | 0.818 | 7.622E-06 | | hypothetical protein, Xrp4 |
| ZP_02243997 | 0.350 | | 3.098E-06 | 0.737 | 7.094E-06 | | hypothetical protein, Xrp11 |
| ZP_02244823 | 0.548 | | 5.026E-06 | 0.700 | 3.152E-06 | | hypothetical protein, Xrp12 |
| ZP_02241620 | 0.257 | | 2.462E-06 | 0.627 | 4.018E-05 | | hypothetical protein, Xrp10 |
| ZP_02243623 | 0.574 | | 3.788E-06 | 0.571 | 1.665E-06 | | hypothetical protein |
| ZP_02243971 | 2.153 | | 1.241E-05 | 0.570 | 4.618E-06 | | hypothetical protein |
| ZP_02243334 | 0.578 | | 4.209E-06 | 0.569 | 1.024E-06 | | hypothetical protein |
| ZP_02244348 | 1.207 | | 4.862E-06 | 0.568 | 6.897E-06 | | hypothetical protein |
| ZP_02245403 | 1.459 | | 9.926E-07 | 0.566 | 5.517E-06 | | hypothetical protein |
| ZP_02244112 | 0.574 | | 1.334E-06 | 0.565 | 3.353E-06 | | hypothetical protein |
| ZP_02242299 | 0.923 | | 2.631E-04 | 0.563 | 7.327E-06 | | hypothetical protein |
| ZP_02242734 | 0.572 | | 4.427E-06 | 0.563 | 1.464E-05 | | hypothetical protein |
| ZP_02245437 | 0.749 | | 2.962E-06 | 0.562 | 1.284E-05 | | hypothetical protein |
| ZP_02241289 | 1.386 | | 1.597E-05 | 0.557 | 6.234E-06 | | hypothetical protein |
| ZP_02243086 | 0.366 | | 5.219E-06 | 0.556 | 1.071E-07 | | hypothetical protein |
| ZP_02244928 | 0.407 | | 3.749E-06 | 0.555 | 3.649E-06 | | hypothetical protein |
| ZP_02241718 | 0.559 | | 1.430E-06 | 0.550 | 3.190E-06 | | hypothetical protein |
| ZP_02244901 | 0.525 | | 3.350E-06 | 0.550 | 2.836E-05 | | hypothetical protein |
| ZP_02242226 | 0.416 | | 1.173E-05 | 0.549 | 7.709E-06 | | hypothetical protein |
| ZP_02241660 | 0.803 | | 3.726E-06 | 0.549 | 1.123E-05 | | hypothetical protein |
| ZP_02244115 | 0.493 | | 7.067E-06 | 0.548 | 1.470E-06 | | hypothetical protein |
| ZP_02241501 | 0.735 | | 2.454E-06 | 0.547 | 2.442E-06 | | hypothetical protein |
| ZP_02245447 | 0.534 | | 6.311E-06 | 0.546 | 1.861E-05 | | hypothetical protein |
| ZP_02241722 | 0.586 | | 3.569E-06 | 0.544 | 5.316E-06 | | hypothetical protein |
| ZP_02244993 | 0.440 | | 1.838E-05 | 0.544 | 5.095E-06 | | hypothetical protein |
| ZP_02245122 | 0.875 | | 9.885E-06 | 0.543 | 1.084E-05 | | hypothetical protein |
| ZP_02243396 | 0.548 | | 2.890E-07 | 0.541 | 5.205E-06 | | hypothetical protein |
| ZP_02243968 | 0.548 | | 6.298E-06 | 0.539 | 5.212E-06 | | hypothetical protein |
| ZP_02242924 | 0.544 | | 6.951E-06 | 0.535 | 3.649E-06 | | hypothetical protein |
| ZP_02243604 | 2.614 | | 2.220E-06 | 0.535 | 2.374E-06 | | hypothetical protein |
| ZP_02242706 | 0.881 | | 8.000E-06 | 0.534 | 3.195E-06 | | hypothetical protein |
| ZP_02243109 | 0.727 | | 2.266E-05 | 0.532 | 2.652E-09 | | hypothetical protein |
| ZP_02244828 | 3.173 | | 8.598E-06 | 0.531 | 2.163E-06 | | hypothetical protein |
| ZP_02241849 | 1.388 | | 8.598E-06 | 0.530 | 3.249E-04 | | hypothetical protein |
| ZP_02244988 | 0.481 | | 5.803E-06 | 0.530 | 3.483E-06 | | hypothetical protein |
| ZP_02243371 | 0.526 | | 3.101E-06 | 0.528 | 2.442E-06 | | hypothetical protein |
| ZP_02242621 | 1.900 | | 1.099E-05 | 0.528 | 8.067E-07 | | hypothetical protein |
| ZP_02242446 | 0.729 | | 1.411E-05 | 0.525 | 4.797E-06 | | hypothetical protein |
| ZP_02244425 | 0.655 | | 6.067E-06 | 0.525 | 3.465E-06 | | hypothetical protein |
| ZP_02242186 | 0.439 | | 3.290E-06 | 0.524 | 5.472E-06 | | hypothetical protein |
| ZP_02241603 | 1.185 | | 1.159E-05 | 0.521 | 9.121E-07 | | hypothetical protein |
| ZP_02244833 | 1.528 | | 1.035E-05 | 0.520 | 1.224E-06 | | hypothetical protein |
| ZP_02245162 | 0.561 | | 4.467E-06 | 0.520 | 3.649E-06 | | hypothetical protein |
| ZP_02242456 | 0.908 | | 2.831E-06 | 0.520 | 3.125E-06 | | hypothetical protein |
| ZP_02243324 | 0.507 | | 4.144E-06 | 0.518 | 1.388E-06 | | hypothetical protein |
| ZP_02244007 | 0.525 | | 2.841E-06 | 0.517 | 5.914E-06 | | hypothetical protein |
| ZP_02244812 | 0.525 | | 1.914E-06 | 0.516 | 2.639E-06 | | hypothetical protein |
| ZP_02243420 | 0.522 | | 6.073E-06 | 0.514 | 5.860E-06 | | hypothetical protein |
| ZP_02242229 | 0.599 | | 1.100E-05 | 0.513 | 3.936E-06 | | hypothetical protein |
| ZP_02244288 | 1.145 | | 1.068E-05 | 0.512 | 7.327E-06 | | hypothetical protein |
| ZP_02241691 | 0.425 | | 2.129E-06 | 0.511 | 1.464E-05 | | hypothetical protein |
| ZP_02243321 | 0.311 | | 3.294E-06 | 0.510 | 1.071E-07 | | hypothetical protein |
| ZP_02243905 | 0.369 | | 6.199E-06 | 0.509 | 8.207E-06 | | hypothetical protein |
| ZP_02242563 | 1.367 | | 3.353E-06 | 0.509 | 1.160E-05 | | hypothetical protein |
| ZP_02245247 | 0.467 | | 1.284E-05 | 0.501 | 8.124E-06 | | hypothetical protein |
| ZP_02244368 | 0.354 | | 6.234E-06 | 0.499 | 5.662E-06 | | hypothetical protein |
| ZP_02243895 | 0.178 | | 3.649E-06 | 0.498 | 3.716E-06 | | hypothetical protein |
| ZP_02245352 | 0.194 | | 3.353E-06 | 0.493 | 4.192E-05 | | hypothetical protein |
| ZP_02243014 | 0.790 | | 7.327E-06 | 0.492 | 1.458E-05 | | hypothetical protein |
| ZP_02242055 | 0.499 | | 1.464E-05 | 0.491 | 4.760E-06 | | hypothetical protein |
| ZP_02243750 | 0.211 | | 1.284E-05 | 0.490 | 9.875E-06 | | hypothetical protein |
| ZP_02244631 | 0.482 | | 6.234E-06 | 0.488 | 1.861E-05 | | hypothetical protein |
| ZP_02243603 | 1.001 | | 1.071E-07 | 0.486 | 5.316E-06 | | hypothetical protein |
| ZP_02243347 | 1.137 | | 9.049E-03 | 0.486 | 3.095E-05 | | hypothetical protein, Xrp8 |
| ZP_02245205 | 0.811 | | 3.353E-06 | 0.484 | 5.212E-03 | | hypothetical protein |
| ZP_02244010 | 0.723 | | 1.464E-05 | 0.483 | 1.152E-05 | | hypothetical protein |
| ZP_02241305 | 1.238 | | 1.284E-05 | 0.479 | 6.929E-06 | | hypothetical protein |
| ZP_02241978 | 0.387 | | 6.234E-06 | 0.475 | 3.649E-06 | | hypothetical protein |
| ZP_02241882 | 0.597 | | 1.071E-07 | 0.474 | 9.018E-06 | | hypothetical protein |
| ZP_02242522 | 0.452 | | 1.235E-05 | 0.472 | 1.130E-05 | | hypothetical protein |
| ZP_02244798 | 2.646 | | 3.190E-06 | 0.472 | 1.111E-05 | | hypothetical protein |
| ZP_02244005 | 1.272 | | 2.836E-05 | 0.470 | 9.511E-06 | | hypothetical protein |
| ZP_02242398 | 0.477 | | 7.709E-06 | 0.469 | 1.151E-05 | | hypothetical protein |
| ZP_02243712 | 0.475 | | 1.123E-05 | 0.468 | 9.957E-06 | | hypothetical protein |
| ZP_02241280 | 0.472 | | 1.470E-06 | 0.465 | 9.875E-06 | | hypothetical protein |
| ZP_02245376 | 0.295 | | 2.442E-06 | 0.462 | 8.067E-07 | | hypothetical protein |
| ZP_02241608 | 2.147 | | 1.235E-05 | 0.462 | 4.797E-06 | | hypothetical protein |
| ZP_02244927 | 0.465 | | 3.190E-06 | 0.458 | 3.465E-06 | | hypothetical protein |
| ZP_02244389 | 0.435 | | 2.836E-05 | 0.457 | 1.224E-06 | | hypothetical protein |
| ZP_02245213 | 0.823 | | 7.709E-06 | 0.455 | 2.646E-06 | | hypothetical protein |
| ZP_02244287 | 0.458 | | 1.123E-05 | 0.451 | 4.648E-06 | | hypothetical protein |
| ZP_02241175 | 0.479 | | 1.470E-06 | 0.447 | 6.199E-06 | | hypothetical protein |
| ZP_02244122 | 0.368 | | 2.442E-06 | 0.443 | 3.649E-06 | | hypothetical protein |
| ZP_02244897 | 0.525 | | 1.235E-05 | 0.441 | 3.812E-06 | | hypothetical protein |
| ZP_02243345 | 1.353 | | 3.190E-06 | 0.439 | 1.665E-03 | | hypothetical protein |
| ZP_02245103 | 0.247 | | 2.836E-05 | 0.438 | 4.618E-06 | | hypothetical protein |
| ZP_02243066 | 0.443 | | 7.709E-06 | 0.436 | 1.024E-06 | | hypothetical protein |
| ZP_02245405 | 0.654 | | 1.123E-05 | 0.436 | 6.897E-06 | | hypothetical protein |
| ZP_02244417 | 0.535 | | 1.470E-06 | 0.434 | 1.854E-05 | | hypothetical protein |
| ZP_02243708 | 0.436 | | 2.442E-06 | 0.429 | 5.517E-06 | | hypothetical protein |
| ZP_02242261 | 0.586 | | 1.235E-05 | 0.428 | 9.875E-06 | | hypothetical protein |
| ZP_02244168 | 0.162 | | 3.190E-06 | 0.428 | 8.067E-07 | | hypothetical protein |
| ZP_02241208 | 0.432 | | 7.709E-06 | 0.425 | 3.465E-06 | | hypothetical protein |
| ZP_02241681 | 0.432 | | 1.123E-05 | 0.425 | 1.224E-06 | | hypothetical protein |
| ZP_02243362 | 0.417 | | 1.470E-06 | 0.410 | 2.646E-06 | | hypothetical protein |
| ZP_02241905 | 0.180 | | 2.442E-06 | 0.409 | 4.648E-06 | | hypothetical protein |
| ZP_02245379 | 0.383 | | 1.235E-05 | 0.402 | 6.199E-06 | | hypothetical protein |
| ZP_02243461 | 0.590 | | 3.190E-06 | 0.390 | 3.649E-06 | | hypothetical protein |
| ZP_02245454 | 0.389 | | 7.709E-06 | 0.383 | 1.665E-06 | | hypothetical protein |
| ZP_02242974 | 0.385 | | 1.123E-05 | 0.379 | 4.618E-06 | | hypothetical protein |
| ZP_02242578 | 0.832 | | 1.470E-06 | 0.374 | 1.024E-06 | | hypothetical protein |
| ZP_02242386 | 0.822 | | 2.442E-06 | 0.370 | 6.897E-06 | | hypothetical protein |
| ZP_02243409 | 0.935 | | 3.190E-06 | 0.360 | 5.517E-06 | | hypothetical protein |
| ZP_02242230 | 0.445 | | 2.836E-05 | 0.357 | 9.875E-06 | | hypothetical protein |
| ZP_02242401 | 0.358 | | 1.123E-05 | 0.352 | 4.797E-06 | | hypothetical protein |
| ZP_02245307 | 0.335 | | 1.470E-08 | 0.330 | 3.465E-06 | | hypothetical protein |
| ZP_02243866 | 0.558 | | 2.442E-06 | 0.321 | 1.224E-06 | | hypothetical protein |
| ZP_02245363 | 0.282 | | 5.410E-06 | 0.315 | 3.246E-06 | | hypothetical protein, Xrp2 |
| ZP_02244277 | 2.537 | | 5.032E-05 | 0.310 | 3.048E-06 | | hypothetical protein, Xrp6 |
| ZP_02245384 | 0.174 | | 5.095E-06 | 0.310 | 6.199E-06 | | hypothetical protein |
| ZP_02244286 | 0.315 | | 1.084E-05 | 0.310 | 3.649E-06 | | hypothetical protein |
| ZP_02242137 | 0.281 | | 5.212E-06 | 0.277 | 1.665E-06 | | hypothetical protein |
| ZP_02244728 | 0.876 | | 3.649E-06 | 0.272 | 4.618E-06 | | hypothetical protein |
| ZP_02244371 | 0.274 | | 2.374E-06 | 0.270 | 1.024E-06 | | hypothetical protein |
| ZP_02244249 | 0.428 | | 3.195E-06 | 0.239 | 6.897E-06 | | hypothetical protein |
| ZP_02242349 | 0.243 | | 2.652E-06 | 0.239 | 1.854E-05 | | hypothetical protein |
| ZP_02241182 | 0.233 | | 2.549E-06 | 0.229 | 2.275E-06 | | hypothetical protein, Xrp14 |
| ZP_02244824 | 1.260 | | 2.442E-06 | 0.216 | 1.438E-06 | | hypothetical protein |
| ZP_02242828 | 0.222 | | 8.067E-07 | 0.201 | 2.218E-06 | | hypothetical protein |
| ZP_02241877 | 0.695 | | 6.097E-06 | 0.188 | 2.124E-06 | | hypothetical protein, Xrp13 |
| ZP_02242303 | 0.106 | | 3.465E-06 | 0.175 | 1.166E-06 | | hypothetical protein |
| ZP_02243912 | 0.189 | | 5.472E-06 | 0.160 | 1.065E-06 | | hypothetical protein |
| ZP_02244133 | 0.084 | | 9.121E-07 | 0.150 | 9.981E-07 | | hypotethical protein |
| ZP_02242886 | 0.151 | | 1.224E-06 | 0.134 | 8.949E-07 | | hypothetical protein |
| ZP_02242871 | 0.064 | | 1.388E-06 | 0.063 | 1.423E-06 | | hypothetical protein, Xrp9 |
| ZP_02241427 | 0.068 | | 5.914E-06 | 0.062 | 6.158E-06 | | hypothetical protein |
| ZP_02244120 | 0.032 | | 5.860E-06 | 0.032 | 2.110E-07 | | hypothetical protein |
| ZP_02242561 | 0.150 | | 1.642E-06 | 0.011 | 2.074E-06 | | hypothetical protein, Xrp1 |
| **Hrp protein** | | | | | | | |
| ZP_02245224 | 0.806 | 6.02E-06 | | 1.34 | 5.36E-06 | | HrcC |
| ZP_02245246 | 0.73 | 6.15E-06 | | 1.157 | 4.63E-06 | | Hpa3 |
| ZP_02245240 | 0.672 | 8.39E-06 | | 1.114 | 8.93E-06 | | HrpD5 |
| ZP_02245229 | 0.519 | 9.75E-06 | | 0.902 | 4.01E-04 | | HrpB4 |
| ZP_02245234 | 0.904 | 2.38E-06 | | 0.852 | 3.41E-06 | | HrcV |
| ZP_02245249 | 0.776 | 1.03E-06 | | 0.652 | 8.15E-06 | | HrpF |
| ZP_02245239 | 0.701 | 6.07E-06 | | 0.641 | 8.02E-06 | | HpaA |
| ZP_02245244 | 0.2 | 2.77E-06 | | 0.596 | 7.45E-06 | | HrpE3 |
| ZP_02245225 | 1.154 | 6.93E-06 | | 0.592 | 4.37E-06 | | HrcT |
| ZP_02245236 | 0.566 | 3.72E-06 | | 0.565 | 9.88E-06 | | HrpD1 |
| ZP_02245245 | 0.562 | 1.74E-05 | | 0.552 | 1.24E-05 | | Hpa4 |
| ZP_02245226 | 0.825 | 8.83E-06 | | 0.521 | 4.08E-06 | | HrpB7 |
| ZP_02245222 | 0.606 | 8.58E-06 | | 0.513 | 2.44E-06 | | Hpa2 |
| ZP_02245241 | 0.51 | 1.77E-06 | | 0.512 | 3.35E-06 | | HrpD6 |
| ZP_02245237 | 0.381 | 7.33E-06 | | 0.508 | 8.78E-06 | | HrcR |
| ZP_02245235 | 0.718 | 1.07E-07 | | 0.499 | 2.81E-06 | | HrpC3 |
| ZP_02245243 | 0.516 | 3.65E-06 | | 0.474 | 9.41E-06 | | HpaB |
| ZP_02245238 | 0.374 | 2.84E-05 | | 0.427 | 4.80E-06 | | HrcS |
| ZP_02245242 | 0.325 | 5.21E-06 | | 0.301 | 3.81E-06 | | HrpE |
| ZP_02245230 | 0.309 | 2.16E-06 | | 0.238 | 5.52E-06 | | HrpB3 |
| ZP_02245231 | 0.036 | 3.48E-06 | | 0.223 | 1.48E-06 | | HrpB2 |
| ZP_02245233 | 0.113 | 3.13E-06 | | 0.111 | 7.41E-07 | | HrcU |
| ZP_02245223 | 0.033 | 2.64E-06 | | 0.049 | 4.91E-06 | | Hpa1 |
| ZP_02245232 | 0.008 | 3.94E-06 | | 0.018 | 1.12E-06 | | HrpB1 |
| **T3S Effectors** | | | | | | | |
| ZP_02241198 | 1.216 | 6.77E-07 | | 1.117 | 4.47E-06 | | XopQ |
| ZP_02242252 | 1.702 | 7.25E-06 | | 1.069 | 4.28E-06 | | XopC2 |
| ZP_02244325 | 2.204 | 5.51E-06 | | 1.01 | 9.55E-06 | | XopY |
| ZP_02244748 | 0.121 | 1.06E-05 | | 0.994 | 3.98E-06 | | XopAK |
| ZP_02241120 | 0.732 | 1.07E-05 | | 0.962 | 3.85E-06 | | XopK |
| ZP_02242107 | 0.777 | 2.81E-06 | | 0.944 | 3.77E-06 | | XopO |
| ZP_02241516 | 0.556 | 4.16E-06 | | 0.643 | 3.37E-04 | | XopAF |
| ZP_02241549 | 0.583 | 3.53E-06 | | 0.582 | 9.78E-06 | | XopW |
| ZP_02245250 | 0.791 | 3.02E-06 | | 0.581 | 9.76E-05 | | XopAE |
| ZP_02241686 | 0.708 | 9.07E-06 | | 0.575 | 9.68E-06 | | XopV |
| ZP_02244521 | 0.869 | 2.89E-06 | | 0.553 | 2.41E-05 | | XopAD |
| ZP_02243302 | 0.432 | 1.81E-05 | | 0.536 | 3.98E-05 | | XopZ1 |
| ZP_02241836 | 0.277 | 3.47E-06 | | 0.512 | 8.90E-06 | | XopI |
| ZP_02244859 | 0.176 | 9.34E-04 | | 0.502 | 3.36E-05 | | AvrRxo1 |
| ZP_02241238 | 1.965 | 3.11E-03 | | 0.497 | 8.71E-06 | | AvrBS2 |
| ZP_02242247 | 0.94 | 6.52E-06 | | 0.425 | 7.82E-06 | | XopP |
| ZP_02243404 | 0.686 | 5.10E-06 | | 0.497 | 7.47E-06 | | XopAA |
| ZP_02242620 | 0.806 | 9.51E-06 | | 0.467 | 1.85E-05 | | XopAB |
| ZP_02245383 | 0.281 | 1.24E-05 | | 0.367 | 1.85E-05 | | XopR |
| ZP_02244127 | 0.463 | 7.71E-06 | | 0.356 | 8.07E-07 | | XopL |
| ZP_02241665 | 0.126 | 3.65E-06 | | 0.117 | 7.79E-07 | | XopX |

^a^ Protein IDs were referred from the data (NZ_AAQN01000001.1, GI:94721269) which are old version of the draft genome sequence of *Xoc* BLS256 strain.

^b^ The expression level of a gene in the *hrpG* mutant R*∆hrpG* or in the *hrpX* mutant R*∆hrpX* compared to the wild-type RS105 presents a ratio. *Xoc* strains were co-cultured with rice cells at 25 ℃ for 16 h and then the RNAs of the tested strains were hybridized *in silicon* microarray. Data with a false discovery rate of 5% and a fold-change minimum of 1.75 higher for up-regulation and of 0.55 lower for down-regulation were listed. As the comparison, the *hrp* genes and the known T3SE-encoding genes were also displayed.

^c^ HrpX-regulated proteins (Xrp) were selected for further investigation according to whether there are T3S signals at the N-termini or there are PIP-like boxes in the promoters of their coding genes. The protein IDs for Xrp (old version) were then changed to the IDs annotated in the new version of the complete genome sequence of *Xoc* BLS256 (NZ_AAQN01000001.1, GI:353459993) (see Table 1).

**Figure S1.** Detection of the expression of two genes encoding T3SEs, *hpa1 and XOC_0618* (*XopX*) in *X. oryzae* pv. *oryzicola* strains which interacted with rice cells.


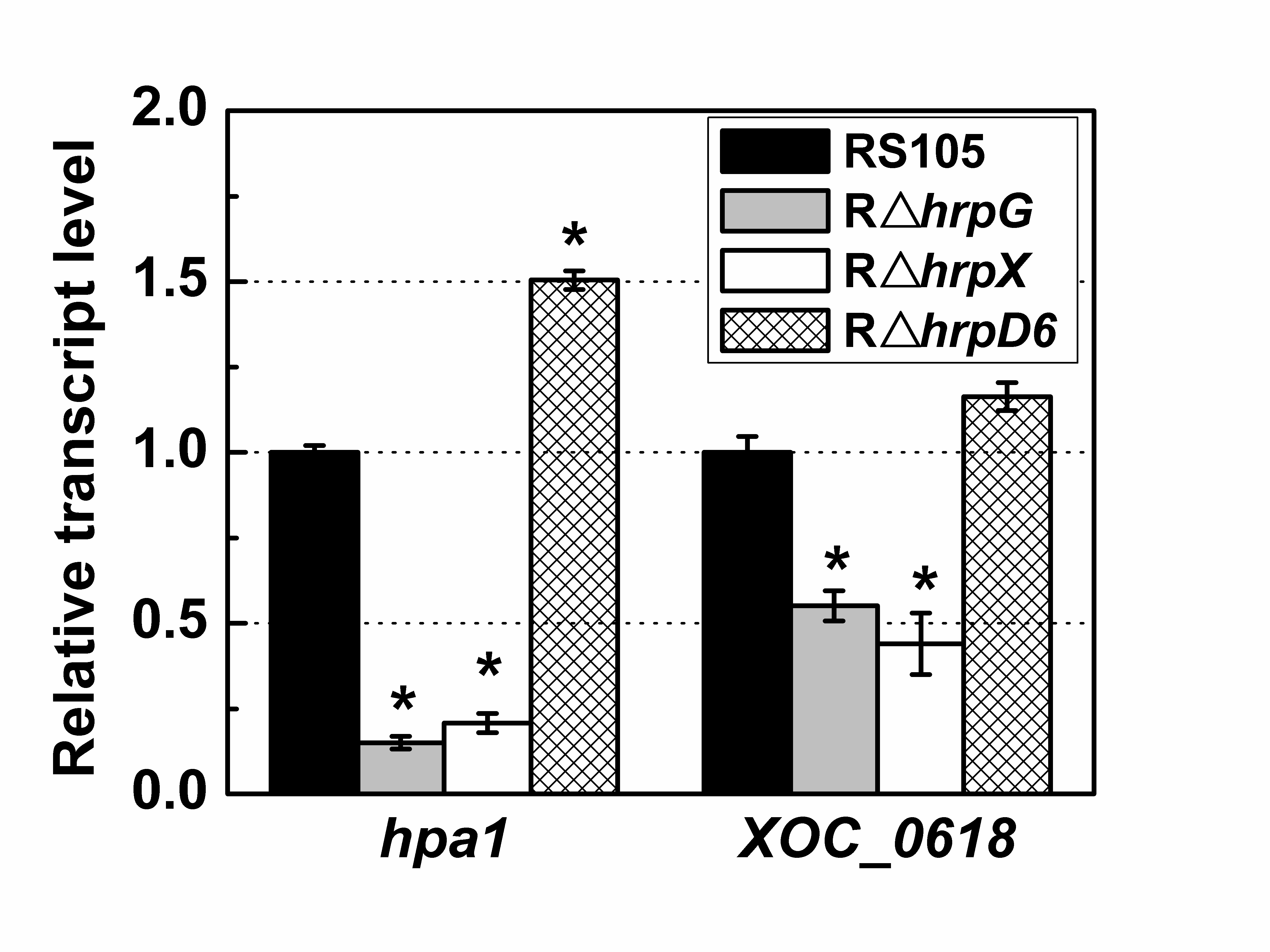


**Figure S1.** Expression of *hpa1 and XOC_0618* detected by qRT-PCR. *hpa1 and XOC_0618* (*xopX*) were identified as T3SEs that are positively regulated by HrpG and HrpX in *X. oryzae* pv. *oryzicola*, but negatively regulated by HrpD6. Data are the mean ± SD of triplicate measurements from a representative experiment; and similar results were obtained in two other independent experiments. The asterisk in each horizontal column indicates significant differences using the Student’s *t* test (**P* < 0.05).
